# MES-FPMIPv6: MIH-Enabled and enhanced secure Fast Proxy Mobile IPv6 handover protocol for 5G networks$\hat{*}$

**DOI:** 10.1371/journal.pone.0262696

**Published:** 2022-05-26

**Authors:** Fikadu Degefa, Jihyeon Ryu, Hyoungshick Kim, Dongho Won

**Affiliations:** 1 Department of Electrical and Computer Engineering, Sungkyunkwan University, Suwon, Republic of Korea; 2 Department of Software, Sungkyunkwan University, Suwon, Republic of Korea; Xidian University, CHINA

## Abstract

Fast Proxy Mobile IPv6 (FPMIPv6) is an extension of the PMIPv6 mobility management deployed as part of the next-generation internet protocol. It allows location-independent routing of IP datagrams, based on local mobility to IPv6 hosts without involvement of stations in the IP address signaling. A mobile node keeps its IP address constant as it moves from link to link, which avoids signaling overhead and latency associated with changing IP address. Even though local mobility requirements hold, it entails security threats such as Mobile Node, Mobile Access Gateway, as well as Local Mobility Anchor impersonation that go beyond those already exist in IPv6. As mobile station keeps moving across different serving networks, its IP remains constant during handover, and location privacy may not also be preserved. Moreover, homogeneous network dependence of PMIPv6 is one of the gaps, which FPMIPv6 could not mitigate. FPMIPv6 does not support heterogeneous network handover, for which numerous researchers have proposed Media Independent Handover (MIH) enabled FPMIPv6 schemes to allow fast handover among heterogeneous networks, but in the absence of security solutions. As a comprehensive solution, we propose a new handover authentication scheme and a key agreement protocol for the ‘MIH-enabled Network Only FPMIPv6’ model. As one of the basic requirements, mobility management should minimize signaling overhead, handover delay and power consumption of the mobile node. The proposed scheme improves wireless link overhead (mobile node overhead) by 6-86% as cell radius, wireless failure probability and number of hop vary. The security of the proposed scheme has also been analyzed under BAN logic and Automated Validation of Internet Security Protocols and Applications (AVISPA) tool and its performance has numerically been evaluated through a pre-determined performance matrix and found to be effective and preferably applicable compared with other schemes.

## 1 Introduction

Mobile wireless communication technologies have evolved rapidly over the past two decades. Quality of Experience (QoE) and quality of service (QOS) required for mobile communication systems led to rapid developments in mobile wireless communication technologies as a driving factor for the emerged innovative wireless networks. These networks are interconnected and inter-operate to deliver internet and other communication services for mobile users anytime anywhere.

Recently, as a result of the diversified services and technologies, most mobile gadgets deployed multiple wireless interfaces to support various wireless technologies such WiFi, WiMAX, UTMS and LTE [1]. Considering that more and more mobile devices have equipped multiple network interfaces, it is important for mobile users to select the most appropriate network interface even multiple interfaces to increase bandwidth and reduce cost. To provide anytime anywhere wireless connection to mobile users, the next generation wireless networks (NGWN) are moving to become all IP-based networks to support ubiquitous wireless environment by interconnecting different wireless access technologies in a heterogeneous infrastructure [1].

In heterogeneous network environment, due to the limited coverage, vertical handover(handover among heterogeneous serving network) frequently occurs when users move from one wireless network to another. Mainly, for 5G ultra dense networks, keeping a continuous on going session during handover is a fundamental operational requirement of quality of service [2]. i.e Efficient mobility management solutions are in need. As the heterogeneous networks become larger and users demand for higher data traffic with quality of services, the mobility management solutions has increasingly become an important area of interest to provide seamless handover mechanisms not only for simple mobile data, but also for real-time and multimedia applications such as voice over IP (VoIP), video conferencing, IPTV, and internet gaming [3].

To provide mobility management services, various solutions have been proposed [4, 5] for the emerging mobile devices, of which IP mobility management solutions have got more attention and many schemes have been proposed [6]. Among these solutions, Mobile IPv6 (MIPv6) [7], which was published by IETF, is most cited scheme, in which Home Agent manages registered Mobile Nodes and maintains bi-directional tunnel for each MN that leaves the home network. As a result of high signaling cost due to frequent movements of the mobile node, Hierarchical Mobile IPv6 (HMIPv6) [8] was proposed to divide a network topology into different domains to reduce the signaling overhead for micro-mobility scenarios, which introduces Mobility Anchor Point (MAP) as the Home Agent of the given domain so that MN’s updating cost can be reduced. Another important enhancement is the Mobile IPv6 Fast Handovers (FMIPv6) [9], which improves handover performance through link layer triggering and pre-registration methods. In particular, FMIPv6 sets up a temporal tunnel between previous and new attachment points to forward buffered data which reduces packet loss. All the above solutions require an involvement of MN which may result in excessive resource consumption for resource-limited mobile devices and needs to change protocol stack. As a result, network-based mobility management, Proxy MIPv6 (PMIPv6) [10–13] has been proposed in order to overcome the host-based limitations, which supports mobility management without of involvement of the mobile. This introduces new entities, namely, Mobile Access Gateway (MAG) and Local Mobility Anchor (LMA), which manages mobility on the behalf of MNs so that handover delay and signaling cost can be reduced as compared with the previous solutions [14, 15]. PMIPv6 has further been improved to Fast handover PMIPv6 (FPMIPv6) [13].

In the context of multiple interfaces of MN and multiple radio access technologies, due to the heterogeneity of such networks, vertical handover is a complex procedure requiring comprehensive standards to facilitate seamless handover between diverse access networks and to inter-operated with multiple mobility management mechanisms. The IEEE 802.21 addresses this issue by providing a media independent handover (MIH) framework which identifies the services and structure to enable seamless handover mechanism in heterogeneous wireless networks [16]. The ultimate purpose of IEEE 802.21 MIH is to provide mobility mechanisms independent of media by offering useful information about link layer and candidate networks [11, 17].

IEEE 802.21 defines a media-independent framework that provides a generic interface between different link layer technologies and upper layers. Link layer technologies include media types specified by the Third Generation (3G) Partnership Project (3GPP), 3G Partnership Project 2 (3GPP2), and both wired and wireless media in the IEEE 802 family of standards. The MIH standard aims to facilitate the integration of heterogeneous networks by providing a uniform information about layer 2 (L2) triggers to the upper layers in order to help the handover decisions. In addition, MIH provides services for inter-technology candidate network discovery, target network selection, and L2 handover initiation and execution [18]. Note that the 802.21 standard does neither specify rules (or policies) for handover decision nor determines whether the handover has to be host-based.

### 1.1 Overview of FPMIPv6

The core idea of Fast Proxy Mobile Internet Protocol Version Six (FPMIPv6), which is a Network based local mobility management architecture, is that the mobile node is not involved in any IP layer mobility-related signalling [19–24]. The mobility management architecture supports movement of IPv6 mobile nodes locally with in a domain without requiring mobility support in the network stack of mobile node. A mobile node keeps its IP address constant as it moves from link to link, avoiding signalling overhead and latency associated with changing IP address. Because mobility is managed by the network on the behalf of the mobile node, specifically software for localized mobility management is not required on the mobile node, whereas IP-layer movement detection software may be necessary, and driver software for link-layer mobility is mandatory. “IP mobility for nodes that have mobile IP client functionality in the IPv6 stack as well as those nodes that do not, would be supported by enabling Proxy Mobile IPv6 protocol functionality in the network” [25]. The core functional entities of this protocol are Local Mobility Anchor (LMA) and Mobility Access Gateway (MAG). MAG performs the mobility-related signalling on behalf of the mobile nodes attached to its access links. It is usually the access router for the mobile mode, that is, the first-hop router in the Localized Mobility Management infrastructure. The responsibility of MAG is tracking mobile node in the local mobility domain (LMD) and detecting of mobile nodes inter into access network and out from access link; it initiates the binding registrations to LMA. LMA within the core network maintains a collection of routes for each mobile node connected to the LMD. The routes point to MAGs managing the links where the mobile nodes are currently located. Packets sent or received to or from the mobile node are routed through tunnels between the LMA and the corresponding MAG. The LMA is a topological anchor point for the addresses assigned to Mobile Nodes in the LMD, which mean packets with those addresses as destination are routed to the LMA.

### 1.2 IEEE 802.21 media independent handover (MIH)

As discussed earlier, IEEE 802.21 working group has built the MIH framework so that upper layers can abstract the heterogeneity aspects of different technologies and interact with them via a unified interface. To handle the particularities of each technology, 802.21 maps this generic interface to a set of media-independent Service Access Points (SAPs) whose purpose is to collect information and control the link behaviors during handovers. In addition, a set of remote interfaces (terminal-network and network-network) are defined to transfer the information stored at the operators’ network to the appropriate locations [13].

**Fig 1:**
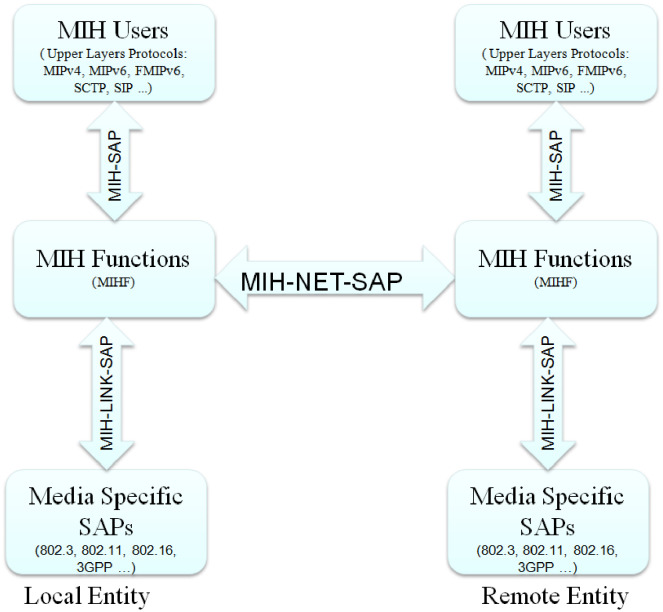
High level media independent handover architecture.

A MIH-enabled node consists a functional entity between link layer and upper layers called Media Independent Handover Function (MIHF). This logical entity functions as an abstraction layer between all upper layers’ mobility management protocols (here called MIH users) and different link layer technologies through media independent interface by obtaining information from lower layers through media specific interfaces(see Fig 1).

Different functional modules have been defined and layered into Service Access Points (SAPs) to provide interface for MIHF to communicate with upper layers (MIH users) and lower layer radio access technologies [26, 27].

Service Access Points (SAPs)—define both media-independent and media-specific interfaces which are intended to access the services provided by each MIH functional entity. Specifically, the SAPs include:

- MIH-SAP, a media independent SAP that provides a uniform interface for higher layers with MIHF, which is particularly defined for MIH-USER to access the services provided by MIHF layer regardless of access technology.
- MIH-LIN-SAP, a media specific SAP interfaces MIHF with media specific links. For the MIHF to provide media independent event service (MIES) and media independent command service (MICS) for a specific link layer technology, media specific SAP (MIH-LINK-SAP) has defined for a specific link layer.
- MIH-NET-SAP, provides the exchange of information between local MIHF and remote MIHF. Particularly, the Media Independent Handover protocol are defined by IEEE 802.21 working group here. The protocol defines message exchange between two MIHF functional entities to provide remote MIH services [28]. Additionally, for the exchange of MIH messages between the local and remote MIHFs, some network communication functions are defined which provide transport services over the data plane.

### 1.3 Integration of FPMIPv6 and MIH

FPMIPv6 is a network layer mobility support solution which provides a fast handover interaction framework and defines the related signaling messages format to reduce handover delay and packets loss. However, to implement and deploy FPMIPv6 in large scale, it should consider the link layer operations and specific access technology. As we take this in to consideration, it still has the following problems: (1)Lack of definition of handover triggers events. For example, FPMIPv6 just gives port message to notify the imminent handover in PMAG-initiated mode, which does not provide the operation in detail. (2) Lack of candidate network discovery and selection mechanism which may result in the handover failure. (3)Lack of handover execution procedure and link-layer specific operations in detail. (4)Lack of detailed explicit heterogeneous handover mechanism [29]. Due to these problems, FPMIPv6 should incorporate other mechanisms such as MIH to support heterogeneous handover.

There are several schemes benefits from the cross layer design of layer 2 (L2) MIH framework and layer 3 (L3) FPMIPv6 protocol to optimize handover latency, packet loss or the overall handover performance [30].

While the basic scenario addressed by PMIPv6 considers MNs with just one interface, [RFC5213] also allows a MN to connect to the same PMIPv6 domain through different interfaces. This limited support of multi-interfaced MNs or heterogeneous handover is not fully specified, since the MAG needs to obtain/guess additional information from the MN, in order to decide whether to treat a MN’s interface attachment as a handover or as new interface attachment (i.e. meaning the creation of a new mobility session and, therefore, the allocation of new home network prefixes to the MN). The use of IEEE 802.21 Media Independent Handover (MIH) Services [IEEE80221] may help in obtaining this additional information. The MIH assisted PMIPv6 protocols enable MAG to deal with multi-technology scenarios [30].

Several schemes have been proposed to integrate the network-based PMIPv6 with MIH framework to optimize the handover performance [31]. These schemes are shown to be fit for heterogeneous network handover and their performance is analytically justified or demonstrated in computer simulation. Despite the fact that the schemes are promising to improve the handover latency, packet loss or seamless communication; there are gaps while considering security perspective of the schemes. These security threats would briefly be discussed under section II of this paper.

As a main objective, it is to briefly discuss security threats of local mobility management protocol and we are concerned to design security solutions. Nevertheless, FPMIPv6 is robust for local mobility management; vulnerabilities in either interface (MAG or LMA interfaces) may entail new security threats that go beyond those already exist in IPv6. Objectives of the potential attacks may be to consume network services at the cost of legitimate mobile node, possibly impersonate the mobile node from a position of off-link, operate under non-existing identity, or cause denial of service to the mobile node or to the localized mobility management domain as a whole [11].

FPMIPv6 does not provide all inclusive security solutions, in particular, it doesn’t have a secure and seamless handover features together. This work extends “HIH-enabled Network Only” handover model to enhance its security so that it holds security and seamless handover requirements in one. The proposed “MES-FPMIPv6: MIH-Enabled and Enhanced Secure Fast Proxy Mobile IPv6 Handover Protocol for 5G Networks” also reduces wireless signaling overhead and handover latency MN between the MAGs, as compare to solutions proposed as of this work. The major contributions of this paper work are summarized as follows:

- Enhancing security solutions and improving performance of handover local mobility for 5G applications. As a security enhanced and performance optimized solution, we propose a scheme that mitigates the identified security breaches of the MIH-enabled FPMIPv6 model.
- Reducing overhead of wireless link of the mobile node during handover by extending and enhancing security of Network Only MIH model.
- We have exhaustively discuss security threats of FMIPv6 with respect to security requirements, for which we proposed solutions and obviously contributes the research of the field.
- We simulated the proposed scheme under Avispa and analyzed under BAN Logic, security protocol verification tools, in order to evaluate the reliability of the protocol, which proves the validity of the scheme and would also contribute to the methods of security protocols verification.
- The security of the protocol is analytically illustrated and a performance of handover and authentication Processes is mathematically demonstrated. As one of the basic requirements, mobility management should minimize signaling overhead, handover delay and power consumption of the mobile node. The proposed scheme improves wireless link overhead (mobile node overhead) by 6–86% as cell radius, wireless failure probability and number of hop vary.

The remaining part of the paper is organized as follows: The paper comes up with brief discussion of problem statements (threats associated with PMIPv6) in the following section II. Section III will be a brief of related researches where as IV, which has different sub titles, will be the security solutions proposed for the identified threats. Section V and VI are security analysis and formal security under protocols verification tools respectively, whereas section VII is performance analysis. VIII is numerical results and comparison and the final section includes conclusion.

## 2 Security threats

Despite its efficiency, Media Independent Handover assisted fast proxy mobile IP version six(MIH-PMIPv6) protocols inherit security threats from PMIPv6/FPMIPv6, which are vulnerable to various attacks.

Most of Mobile IP security threats are through binding update attacks, which result in Denial of Service (DoS), man-in-the-middle, Hijacking, Confidentiality, and Impersonation attacks [12]. The most of the threats are found to be caused by the false binding update to the network, mainly during mobile node handover, for which the security protocol design objective is to make the routing changes secure, including handover and route optimization mechanisms.

The local mobility management protocol is executed on the interface between MAG and LMA as well as between MAG and mobile node (MN). This is to establish, update, and tear down routes both for signaling and data plane traffic of mobile nodes. As this paper is mainly to propose a solution for MIH assisted FPMIPv6 handover security threats in particular, a possible attacks during handover are dictated below as these are basically discussed in [12].

### 2.1 Compromised MAG and LMA

Provided that the mobile node (MN) is to handover from neighbor/candidate network with in a local mobility domain and its handover request is served, a compromised LMA can ignore route binding update from the candidate network’s MAG in order to deny service to MN. In addition to that, a compromised LMA may also direct all handover request to a single MAG or forward all data plane traffics to a single MAG by manipulating its routing table. This may result to denial of service. The compromised MAG may falsely send route binding update request to LMA, pretending that the mobile node has sent the request for handover. Moreover, the compromised MAG may trick a LMA in to believing that large number of MNs has attached to the MAG which results in denial of service. All these threats apply not only to a compromised MAG, but also to an attacker that manages to counterfeit the identity of legitimate MAG in interaction with mobile node and LMA which can be categorized as impersonation.

### 2.2 Impersonated MAG and LMA

An intruder who be able to impersonate these two entities, can forge, modify, or drop route update packets so as to cause an establishment of incorrect routes or the removal of routes that are in active use. It may also consume network services at the cost of legitimate mobile node.

### 2.3 Impersonated Mobile Node (MN)

An attacker that is able to forge the mobile node identity of a mobile node can trick a MAG into believing there is a handover request from a legitimate user or redirecting data plane packets for the mobile node to the attacker. The attacker can launch such an impersonation attack against a mobile node that resides on the same access link with the attacker, or against a mobile node on a different link. If the attack is on the same link with attacker, there would be no route update signaling between MAG and LMA; because the redirection of packets from the mobile node to the attacker is internal to the MAG [30].

Off-link impersonation requires the attacker to fabricate handover signaling of the mobile node and thus trick the MAG into believing that the mobile node has handed over onto the MAG’s access link. The attack is considered to be one of the attacking scenarios if both the attacker and the mobile node are on separate links that connect to different MAGs.

### 2.4 Man-in-the-middle attack

As one of the major attacks for mobile node handover, an attacker that can interpose between a mobile node and a MAG during link- and/or IP-layer Hanover signaling, may be able to launch a man in-the-middle attack on the mobile node by tricking the mobile node into believing that it has a legitimate connection with the localized mobility management domain. This enables the attacker to intercept, inspect, modify, or drop data plane packets from or to the mobile node.

### 2.5 Replay attack

An adversary can resend a handover request message sent earlier from legitimate user in order to use the network free in the expense of legal MN or to impersonate.

### 2.6 Verifier impersonation

The attack that an adversary creates independent connection with the victims and sends messages between them, causing them to think that they are directly talking to each other over local mobility domain while indeed the whole conversation is manipulated by the attacker [32].

### 2.7 Server stolen-verifier

Server stolen-verifier attack: if the authentication server stores the MN’s verification table, the authentication scheme would be vulnerable to stolen-verifier attacks. An intruder could forge a valid identity after somehow stealing the stored verifier.

### 2.8 Location privacy

Keeping the MN’s IP address or Mobile node identity fixed and easily accessible over the FPMIPv6 domain, during initial connection attachment or when executing handover procedure, may result in MN’s location privacy unprotected.

## 3 Related works

When we refer to RFC-5213 [10], in PMIPv6 domain, the main entities (LMA and MAG) and the signaling messages such as Proxy Biding Update (PBU) and Proxy Binding Acknowledgment (PBA) exchanged between these two entities are protected under IPsec using the established security association between them. Signaling messages would be protected using Encapsulating Security Payload (ESP) in transport mode with mandatory integrity protection, however confidentiality protection is not considered which is a security gap for different attacks to be launched. Referring to a deployment of IPSec for PMIPv6, it is briefly discussed in [33] that a man-in-the middle and denial of service attacks are some of the potential attacks possibly launched, which is also the same for FPMIPv6.

[34] states that most wireless technologies, such as IEEE 802.11 and IEEE 802.16, adopt an extensible authentication protocol with transport layer security (EAP-TLS) scheme for achieving the mutual authentication, which would also be applied to the PMIPv6/FPMIPv6 networks. The authors figured out that the scheme has two drawbacks. In the first place, there are huge authentication message overhead that exchanged between the MN and the AAA. Secondly, EAP-TLS does not provide the local authentication mechanism, and as a result the AAA has to validate the MN each time the MN attaches to a different MAG. The more the distance between the AAA and the MN is, the longer the authentication latency will be. In addition, EAP-TLS is still susceptible to a malicious MAG attack, DoS attacks, anonymity support for MNs [35].

A secure fast handover mechanism for Proxy Mobile IPv6 networks(SF-PMIPv6) [36], proposed an authentication scheme to reduce authentication delay, signaling cost and handover latency by using piggyback scheme and pre-handover authentication. Additionally, the scheme includes double buffer mechanism to resolve packet loss problem during MN handover. However, the authentication scheme in SF-PMIPv6 has a single point of failure, which based on a single symmetric key shared among the AAA server and all the MAGs. A secure password authentication mechanism for seamless handover(SPAM) [34] is proposed that executes two separate mutual authentications as One is between the MN and the MAG and the other is between the MAG and the LMA. The authors [32, 37] analyzed and discussed that this scheme is vulnerable to the critical attacks such as stolen smart card, off-line dictionary, replay and impersonation. These researchers also discussed that an identity of MN and shared session key between MN and MAG can be leaked which may result in violation of anonymity and confidentiality requirements. It is also shown that smart cards are vulnerable to loss and/or theft, which makes the SPAM scheme susceptible to password guessing attacks.

Dongwoo et al. [32] analyzed SPAM security gaps and came up with enhanced user authentication for proxy mobile IPv6 networks by extending the smart-card and password based authentication approach. This proposal fills most of the security gaps of the SPAM, the authors claim. The scheme incorporates a bio-metric authentication factor as one of the solutions to mitigates the security threats which may result in misuse of limited MN’s resources, computational cost and processing delay. Additionally, the schemes fails to mitigate the compromised MAG and Compromised LMA security threats discussed in [12]. Beside that, as commonly stated, smart cards are not tamper-proof and vulnerable to loss and/or theft.

As a part of securing the local and global mobility management, [38] have proposed a public key based authentication protocol that includes multiple domains handover i.e inter-domain handover and intra-domain handover. In the proposed scheme, all the PMIPv6 network entities and the MNs use certificates to distribute their public keys among themselves rather than relying on the AAA server which obviously needs public key infrastructure. An authentication scheme [39] based on a concept of ticket introduced by Kerberos work group, in which the ticket includes an encrypted authentication key between MN and MAG plus an expiration time, has been proposed. The ticket would be used during MN handover whenever it is with expiration time. In case of this scheme, once the authentication key is exposed, perfect backward and forward Secrecy properties would not be guaranteed. Moreover, it has a fundamental drawback that it needs to interact with the AAA server again for renewal/reprocess of the ticket when goes out of its life time.

Sanaa et al. [40] have proposed an anonymous and location privacy-preserving scheme for mobile IPv6 heterogeneous networks of two sub-scheme. These are: i) Anonymous home binding update to add anonymity and location privacy to mobile IPv6 binding updates. ii) Anonymous return routability to protect the anonymity of return routability control messages. An onion routing is used to encrypt communicated messages at each intermediate node to achieve location privacy of mobile nodes. In an onion routing mechanism, messages are encapsulated in layers of encryption, analogous to layers of an onion. To authenticate a MN to its foreign gateway and to minimize computational cost of the certificate management process, the scheme uses Certificateless Public Key Cryptography (CL-PKC). The utilization of onion routing in the scheme incurs computational cost. On top of that, a susceptibility of onion routing mechanism when adversaries have access to large fractions of its input-output links [41].

Ryu et al. [42] proposed an authentication scheme based on the AAA server, aiming to reduce packet loss during handover. When a serving MAG becomes aware of a MN’s detachment, it sends the PBU message on behalf of candidate MAG to establish a tunnel between LMA and the candidate MAG with in predefined period of time. Because the MN has to interact with the AAA server each time a handover occurs, it causes a packet.

In general, almost all of the schemes discussed as a related works so far have two unfilled gaps in common, in addition to the drawbacks or vulnerabilities assessed with respect to different security requirements. These are:

i) do not consider a scenario of compromised MAG and Compromised LMA that discussed under a research work [12]. ii) None of them is Media Independent Handover (MIH) enabled scheme and their security solutions proposed are not adaptable to MIH-assisted FPMIPv6 heterogeneous networks. As a result, they are less of benefits of Media Independent Handover (MIH) enabled protocols.

To benefit from MIH IEEE 802.21 handover protocol, Vishal et al [43] proposed a security solution for MIH-based F-PMIPv6 cross-layer handover scheme [29]. This paper proposes an MIH-based secure cross-layer handover protocol for Fast Proxy Mobile IPv6 networks (MIH-SPFP) which can support handoffs in highly dynamic and heterogeneous IoT networks empowered by the mobile 5G technology. The scheme could give an answer to the second of drawback discussed above, which the other schemes lack. But, a compromised MAG and Compromised LMA security threats remain as a question here as well.

## 4 Proposed scheme

### 4.1 MIH enabled FPMIPv6 handover model

There are several schemes benefits from the cross layer design of IEEE 802.21 MIH framework and layer 3 (L3) PMIPv6 protocol to optimize handover latency, packet loss or the overall handover performance. These schemes rely on PMIPv6 properties to exploit the MIH services and MIHF capabilities to enhance heterogeneous networks handover. The integration of FPMIPv6 and MIH is classified in to different categories and their operations are detailed using signaling flow diagram in [31].

We (authors of this paper) adapted a PMIPv6 assisted MIH Using MIHF at Network Side Only Handover model for which we have proposed a security solution by improving few of the protocol flow. The main idea of this proposed by handover model is to provide fast handover for the MN regardless of the presence or absence of IP mobility functionality as well as MIH functionality at the MN, which fully maintains the main objective of FPMIPv6 network-based mobility management. i.e Avoiding involvement of MN from local mobility management signaling or related signaling to reduce signaling overhear and power consumption of resource limited MN.

**Fig 2:**
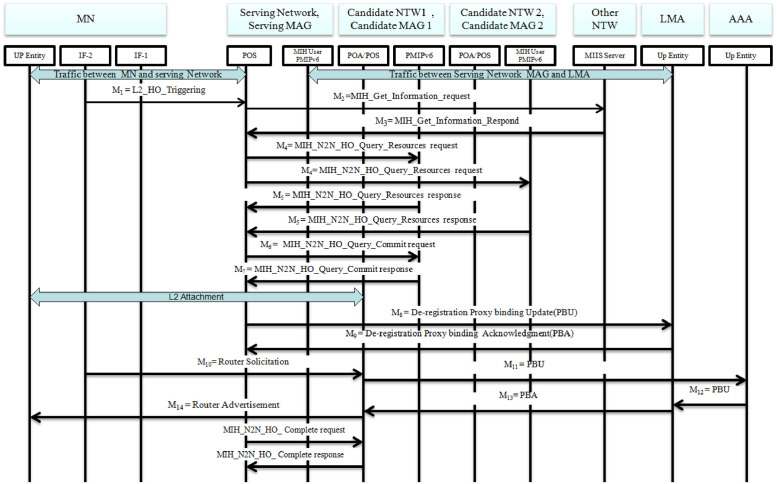
MIH-based PMIPv6 handover scheme.

For the purpose of our security solution, we have amended the MIH-Net-HO-candidate-Query request and MIH-Net-HO-candidate-Query response commands, which would be implicitly managed by the Serving MAG instead of the mobile node. Here is where the change of flow of the protocol has been made. A generalized procedural flow of the MIH based PMIPv6 handover protocol is illustrated in Fig 2 for which its detail will be presented along with proposed security solutions.

### 4.2 Preliminaries and notations

Let G_1_, G_T_ be two cyclic groups of order q. One can say that a map ${ê}_{\text{n}}:G_{1}^{n}\to G_{\text{T}}$ is called a n-linear mapping if it satisfies the following properties:

1. If a_1_,a_2_,…, a_n_ ∈*Z* and g ∈ *G*_1_, then we have ${\hat{\text{e}}}_{\text{n}}\left(g^{a_{1}},g^{a_{2}},\ldots ,g^{a_{\text{n}}}\right)={\hat{\text{e}}}_{\text{n}}{\left(\text{g},\text{g},\ldots ,g\right)}^{a_{1}a_{2}\ldots a_{\text{n}}}$.
2. If the elements *g* ∈ *G*_1_ are all generators of their groups *G*_1_, then $\hat{e}(g,g,\hat{A}\hat{A}\hat{A},g$) is a generator of *G*_T_.

### 4.3 Pre-shared parameters and assumptions

Pre-shared parameters are considered as security credentials shared with mobile node during subscription or shared among network entities during network planning. These are mainly stored in both authentication center and mobile node in secure manner so that the entities can access during handover request or when authentication is needed for any other procedures. The proposed scheme design assumptions and the parameters that are considered to be shared during subscription or during network planning are generally discussed as follows:

- *K*: a shared private key between mobile node and the home authentication system (AAA server) that obtained during subscription through Extensible Authentication Protocol (EAP), Transport Layer Security (TLS) and Evolved Packet Core-Authentication and Key Agreement (EPS-AKA) for WiFi, WiMAX, and LTE or LTE-A respectively.
- *KI*: newly introduced as unique Key Identifier which is a pointer to the key *K* that has to be changed or re-assigned after each successful execution of handover protocol or after each successful attachment. Assume *KI* is known to both MN and AAA server.
- It is assumed that there is a secret key K*_MAG-LMA_ shared between the LMA and the MAG, which enables establishment of a secure channel between the two entities or allows them to authenticate each other, where * denotes C or S.
- There is an assumption that there would be established secure channels between LMA and AAA server.
- It is also assumed that there is a shared secret key K_MIH-AAA_ between MIH server and AAA server.
- Key derivation functions (KDF) and Crypto algorithms that are cryptographic hash functions that generate one or more secret keys from a secret value such as a main key (K).
- There would be a lookup table for indexed random generated numbers resides at MN and AAA server, which is for freshness of generated keys during handover.

### 4.4 Pre-Handover key agreement

Pre-Handover Key Agreement is executed during network planning or before the mobile node joins FPMIPv6 domain. It is intended to agree on handover authentication keys in advance of handover MN mobility or crossing the territory of candidate MAG so as to reduce latency occurred due to key agreement and a unique a identity assignment to core entities. Unless we have strong and secure handover protocol with secure key agreement and distribution solution, an attacker who is able to forge the identity or who can access the control signal of these two entities can impersonate them to consume the resource in the expense of legitimate nodes or interpose in a mobile node’s communications. Hence, using the keys and the identities shared during pre-handover key agreement, the authors have proposed an authentication protocol so that AAA server (Authentication, Authorization, and accounting server), which is considered to be managed under internet service provider, would authenticate the core entities.

In developing the key agreement protocol, we assume that *n* − 3 number of MAGs, a MIIS server and an LMA have a diameter interaction with the AAA server. As a main objective of the key agreement, MAGs, LMA, MIH-serve and AAA server share group key K_group_. This shared group key shall be used for multi-cast or broadcast media independent handover messages/ commands among MIH supporting entities, which would significantly reduces number of messages sent per handover process. It also functions as mutual authentication key among MAGs and LMA, which is ultimately reduces computational and communication cost that could be incurred due to farther key agreement between each MAG and LMA. A brief procedural discussion of the pre-attachment key agreement steps is detailed as follows, having the preliminary parameters under section 4.2.

1. AAA server publishes cyclic groups G_1_, G_T_ (generators g, g_T_, order q) where Discrete Log (DL) problem is hard, with an efficient *n*-linear map *e* in which (*n* − 2)^*th*^, (*n* − 1)^*th*^ and *n*^*th*^ entities are considered to be LMA, MIH server and AAA server respectively.
2. For *i* = 1, …*n* − 3, *MAG*_i_ chooses *a*_i_ ∈ *Z*_q_, computes $y_{\text{i}}=g^{a_{\text{i}}}$ and broadcasts over the network where AAA also publishes $g^{a_{\text{n}}}$.
3. Subsequently, all MAGs and the servers compute a shared key ${\text{K}}_{\text{group}}={\hat{\text{e}}}_{n}\left(g^{a_{1}},g^{a_{2}},\ldots ,g^{a_{\text{n}}}\right)={\hat{\text{e}}}_{n}{\left(g,g,\ldots ,g\right)}^{a_{1}a_{2}\ldots a_{\text{n}}}$.
4. AAA server generates a sequence of number, which symbolized as *SQN* in the protocol message flow, and broadcasting it to all group members as it will be used to assure freshness of messages broadcasting among the group during handover.

In general, these procedures are said to be pre-handover key agreement, because they are executed during network planning or before attachment of mobile node to the FPMIPv6 domain and also considered as preparation phase for the handover process in which delay due to the key agreement would be reduced.

#### 4.4.1 Secure MIH enabled PMIPv6 handover protocol

**Fig 3:**
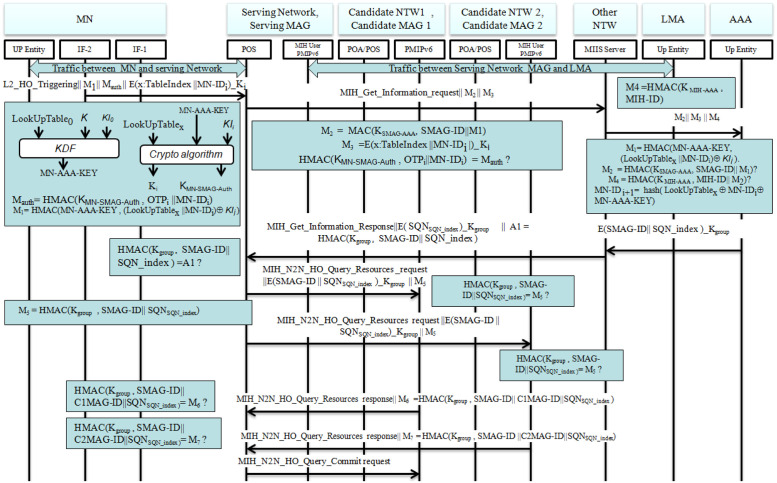
Secure MIH enabled PMIPv6 handover protocol.

**Fig 4:**
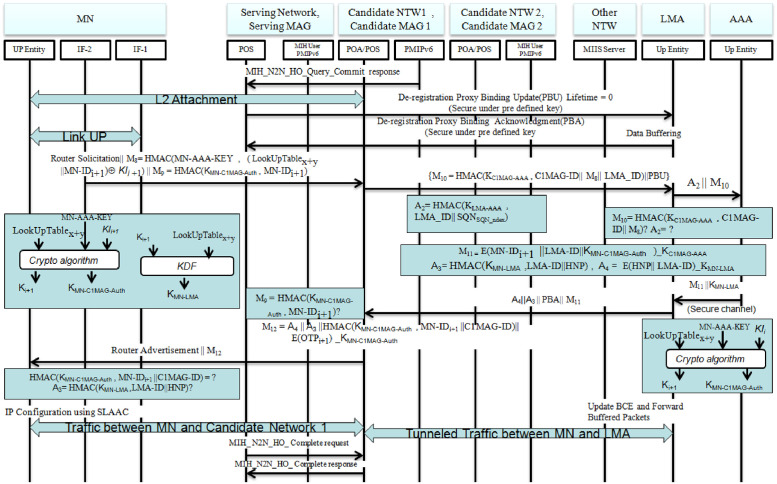
Secure MIH enabled PMIPv6 handover protocol (continued).

As it has been already covered; the mobile node, MAG and LMA are the parties that are vulnerable to threats due to local mobility of the node with in a domain. While the mobile node crosses a boundary of serving network, it would be authenticated to new network for service access. The mobility entities are also authenticated by the server through executing procedures that are defined under this section using predetermined parameters and keys the entities agree on. Moreover, they themselves authenticate each other on identities received from AAA. The proposed protocol (Figs 3 & 4) covers mutual authentication of MN, MAG and LMA during handover by ensuring the overall secure flow of control plane signalling and challenging the parties mainly through Message Authentication Code verification method. Information gathering, resource checking, resources preparation, and resource release are core handover processes for which Media Independent Handover (MIH) protocol is mainly needed to integrate with PMIPv6 local mobility management scheme. While the network performing handover on the behalf of the MN, these processes are executed through MIH commands to fill gaps due to media dependence in heterogeneous networks. Once these are performed via MIH services, the remaining handover procedures are managed through the conventional PMIPv6 handover scheme by adding security solutions as enhancement. To elaborate the procedural execution of the protocol fully, a detailed steps of each operation of the proposed security solution for the local mobility management protocol are presented as follows(see Table 1 for symbol notation description):

1. Handover Triggering: As illustrated in the protocol flow diagram of Fig 3, when the MN sense weak signal strength using its L2 mechanisms, it generates a handover triggering message and informs the serving network. This Handover triggering message is accompanied by security credentials such as predetermined random numbers table index (x:TableIndex), mobile identity obtained during preceding handover or initial attachment, hashed message authentication code through shared key during registration phase (*MN-AAA-KEY*) and key identifier (*KI*). An interface (IF-2) of MN sends the handover triggering message to serving MAG’s Point of Service (POS) that structured as:
  $$L2-HO-Triggering∥M1∥M_{\text{auth}}E\left(x:TableIndex∥MN-ID_{i}\right)\_K_{i},$$
  where M1 = *HMAC*(*MN-AAA-KEY*, (*LookUpTable*_x_ ∥ *MN-ID*_i_) ⊕ *KI*_i_), M_auth_ = *HMAC*(*K*_MN-SMAG-Auth_, *OTP*_i_ ∥ *MN-ID*_i_), *K*_i_ = *Crypto-Alg*-2(*MN-AAA-KEY*, *LookUpTable*_x_ ∥ *KI*_i_) and *K*_MN-SMAG-Auth_ is an authentication key shared between MAG and MN in similar fashion as shown in steps ahead.
2. Information Retrieving Request: After receiving the handover indication message from the MN, the serving network entity (POS), verifies an identity of the MN and sends MIH-Get-Information-request command to Media Independent Information Service (MIIS) server, which is to retrieve information about neighbouring networks through MIH functional modules. MN’s identity is verified by serving MAG through validating its authentication code as:
  *HMAC*(*K*_MN-SMAG-Auth_, *SMAG-ID* ∥ *MN-ID*_i_) = *M*_auth_?
  The message sent to MIIS would be compiled by encrypting serving network MAG identity (*SMAG-ID*), *MN-ID*_i_ and *M*1 through shared key *K*_SMAG-AAA_ between serving network MAG and authentication server, assuming it was agreed on in advance. The signaling message is framed and sent as follows:
  MIH-Get-Information-request∥M2∥M3∥,
  where *M*2 = *HMAC*(*K*_SMAG-AAA_, *SMAG-ID* ∥ *M*1) and M3 = *E*(*x*: *TableIndex* ∥ *SMAG-ID* ∥ *MN-ID*_i_ ∥ *M*1) as M3 is encrypted under the key *K*_SMAG-AAA_.
3. Before responding to the information request of serving network, MIIS server relays the message from MAG to AAA server by adding its own digested identity to verify legitimacy of the mobile node and the MAG so that a network congestion due to falsely generated requests from adversaries or compromised entities would be reduced. The message received by AAA server would be forwarded as:
  M2∥M3∥M4.,
  where M4 = HMAC(K_SMAG-AAA_, SMAG-ID).
4. Identity Verification: Through message authentication mechanism, the AAA server verifies MN’s and MAG’s legitimacy which is mainly depend on secrecy of pre-shared keys among these parties. By verifying *M*2 and *M*4, the authentication server computes new identity (*MN-ID*_x+y_) using *MN-AAA-KEY*, old MN ID (*MN-ID*_i_) and predetermined random number from lookup table for which its index is chosen and sent to the server. The following are equated or computed at this stage:
  $$HMAC(K_{\text{SMAG-AAA}},SMAG-ID∥M1)=M2?$$
  $$HMAC(K_{\text{MIHAAA}},MIH\text{ID}∥M2)=M4?$$
  $$MN-ID_{i+1}=hash\left(LookUpTable_{x}⊕MN-ID_{i}⊕MN-AAA\text{-}KEY\right).$$
  Note that *y* is a predefined number through which the randomness of a number chosen from lookup table is increased or maintained.
5. Provided that there is shared group key *K*_group_, the authentication server/AAA server sends a serving MAG ID and index of sequence of numbers that shared to the parties of the group in advance by encrypting under the shared group key. i.e.
  $$E\left(SMAG-ID∥SQN-index\right)\_K_{\text{group}}.$$
6. Information Retrieving Response:
  The MIIS sends back MIH-Get-Information-Response to serving network MAG which includes neighbouring candidate networks status, *SQN*_SQN-index_ and its message authentication code hashed via group key. The message is framed as:
  $$MIH-Get-Information-Response∥E\left(SQN_{\text{SQN-index}}\right)\_K_{\text{group}}∥$$
  *A*1 = *HMAC*(*K*_group_, *SMAG-ID* ∥ *SQN-index*).
7. Resource Query:
  Up on receiving the Information Retrieving message, the serving MAG equates *HMAC*(*K*_group_, *SMAG-ID* ∥ *SQN-index*) = *A*1?, to verify whether the messenger is really a legitimate entity of the network group or not.
  Consequently, the serving network MAG, specifically POS sends MIH-N2N-HO-Query-Resources-request to the all candidate networks in group to assure availability of resources to host MN in handover process. The request message would be constructed as shown below:
  *MIH-N*2*N-HO-Query-Resources-request* ∥ *E*(*SMAG-ID* ∥ *SQN-index*)_*K*_group_ ∥ *M*5, where *M*5 = *HMAC*(*K*_group_, *SMAG-ID* ∥ *SQN*_SQN-index_).
8. Resource Query Response:
  The candidate networks compute an authentication code *HMAC*(*K*_group_, *SMAG-ID* ∥ *SQN*_SQN-index_) and compare with *M*5 to verify the messenger is a trusted group member. Once that is confirmed, the candidates respond to the resource query by forwarding *MIH-N*2*N-HO-Query-Resources-response* ∥ *M*6 = *HMAC*(*K*_group_, C*MAG-ID ∥ *SQN*_SQN-index_) to the serving network, where * would be *n* − 3 number of MAGs of the candidate networks.
  The serving network MAG authenticates the candidate networks on their digested identities C*MAG-ID as it computes this as:
  $$HMAC(K_{\text{group}},SMAG-ID∥C*MAG-ID∥SQN_{\text{SQN-index}})=M6?$$
  As a last step for L2 attachment to a selected candidate network, the serving network MAG sends MIH-N2N-HO-Query-Commit-request to a candidate network with enough resource capability so that quality of service is maintained. Once the candidate network responds to the query, completion of L2 attachment is guaranteed.
9. De-registration:
  The serving network sends a De-registration Proxy binding Update (PBU) to Local Mobility Anchor (LMA) through pre-established secure channel to de-registered the MN from its network for which LMA sends a De-registration Proxy Binding Acknowledgment (PBA) in response to the PBU. At this step, LMA starts to buffer all data linked to MN from Corresponding Node (CN) which would be forwarded to the MN when L3 handover procedures are finalized.
10. Router solicitation: MN sends a router solicitation to obtain an home network IPv6 prefix (HNP). As one of security credentials to be sent, the mobile node computes a new MN ID, which is dynamic for the sake of maintaining privacy requirements, in the same procedure with AAA server. MN also computes L3 handover procedures (FPMIPv6 procedures) authentication and signaling keys through pre-defined key generation functions and crypto algorithms. These are computed as follows:
  $$\begin{array}{c} MN\text{-}ID_{\text{i}+1}=hash(LookUpTable_{\text{x}}⊕MN\text{-}ID_{\text{i}}⊕MN\text{-}AAA\text{-}KEY).\\ K_{\text{MN-C}1\text{MAG-Auth}}=Crypto\text{-}Alg\text{-}1(LookUpTable_{\text{x}+\text{y}},MN\text{-}AAA\text{-}KEY,KI_{\text{i}+1})\\ K_{\text{i}+1}=Crypto\text{-}Alg\text{-}2(LookUpTable_{\text{x}+\text{y}},MN\text{-}AAA\text{-}KEY,KI_{\text{i}+1})\\ K_{\text{MN-LMA}}=KDF(LookUpTable_{\text{x}+\text{y}},K_{\text{Channel}}) \end{array}$$
  The solicitation signal with authentication codes hashed under the generated new MN-ID and authentication keys is delivered to the candidate MAG(C1MAG) as:
  $$Router-Solicitation∥M8=HMAC(MN-AAA-KEY,(LookUpTable_{x+y}\left.\left.\|MN-ID_{\text{i}+1}\right)⊕KI_{\text{i}+1}\right)\|M9=HMAC\left(K_{\text{MN-C}1\text{MAG-Auth}},MN-ID_{\text{i}+1}\right)$$
11. Proxy binding update (PBU): Receiving the router solicitation request, *C*1*MAG* sends a proxy binding update message
  $$M10=HMAC(K_{C1\text{MAG-AAA}},C1MAG-ID∥M8∥LMA-ID)∥PBU$$
  to LMA.
  Adding its digested ID, i.e *A*_2_ = *HMAC*(*K*_LMA-AAA_, *LMA-ID*)) under pre-shared key, LMA forwards the message to authentication server.
12. Up on receiving PBU signal, AAA server validates MN’s, MAG’s and LMA’s identities through message authentication codes sent as validity accreditation from these entities. As discussed under Security Threats section of this paper, the compromised MAG may trick a LMA in to believing that large number of MNs have attached to the MAG. Here, because AAA verifies the mobile node identity before moving to the next procedure, MAG cannot trick LMA in to believing that large number of MNs have attached to it. AAA server verifies:
  $$HMAC(K_{C1\text{MAG-AAA}},C1MAG-ID∥M8∥LMA-ID)=M10?$$
  $$HMAC(K_{\text{LMA-AAA}},LMA-ID∥SQN_{\text{SQN-index}})=A2?,$$
  where *M*8 = *HMAC*(*MN-AAA-KEY*, (*LookUpTable*_x+y_ ∥ *MN-ID*_i+1_) ⊕ *KI*_i+1_).
  After verifying a legitimacy of MN, candidate MAG and LMA; the authentication sever delivers a computed key *K*_MNLMA_ to LMA via pre-established secure channel. The server sends the key with a message, which is framed as *M*11 ∥ *K*_MN-LMA_ that would be forwarded to the MAG which the MN handover to. *M*10 would be computed as:
  $$M11=E\left(LMA-ID∥K_{\text{MN}-C1\text{MAG-Auth}}∥MN-ID_{i+1}\right)\_K_{C1\text{MAG-AAA}}$$
13. Proxy binding ACK (PBA):
  Once *K*_MNLMA_ and message attached with are received, LMA allocates home network prefix for the MN and creates an entry to binding cache. Subsequently, LMA encrypts HNP and its ID under *K*_MNLMA_ and sends to *C*1*MAG* compiling with *M*11, *PBA* and authentication Message *A*3, which would be constructed as *A*4 ∥ *A*3 ∥ *PBA* ∥ *M*11, for which *A*3 and *A*4 are computed in a way shown below:
  $$A3=HMAC(K_{\text{MN-LMA}},LMA-ID∥HNP)$$
  $$A4=E\left(HNP∥LMA∥ID\right)\_K_{\text{MN-LMA}}$$
  *C*1*MAG* compares the authentication message *M*8 attached to router solicitation request sent from MN with *HMAC* hashed from MN ID using the key *K*_MN-C1MAG-Auth_. i.e
  $$HMAC(K_{\text{MN}-C1\text{MAG-Auth}},MN-ID_{i+1})=M9?$$
  Verifying the identity of MN through the above authentication code, MAG (*C*1*MAG*) send a message *RouterAdvertisement* ∥ *M*12, in response to router solicitation request to MN, where *M*12 = *A*4 ∥ *A*3 ∥ *HMAC*(*K*_MN-C1MAG-Auth_, *MN − ID*_i+1_ ∥ *C*1*MAG-ID*) ∥ *E*(*C*1*MAG-ID*)_*K*_MN-C1MAG-Auth_ ∥ *OTP*_i+1_ as *OTP*_i+1_ will be used for next hop/handover.
14. IP Configuration:
  Up on delivery of the message, MN authenticates MA and LMA through authentication codes received as follows:
  $$HMAC(K_{\text{MN}-C1\text{MAG-Auth}},MN-ID_{i+1}∥C1MAG-ID)=?$$
  $$HMAC(K_{\text{MN-LMA}},LMA-ID-HNP)=A3?$$
  Eventually, router advertisement through which IP address of MN would be configured and traffic tunnelling between the MAG and LMA are executed while the the serving MAG (*SMAG*) sends *MIH-N*2*N-HO-Complete-request* in order to complete the handover process.

**Table 1:** Notations.

Notations	Descriptions
M_i_	*i*^*th*^ message of the handover protocol message flow
MN-ID_i_	*i*^*th*^ mobile node identity as it is dynamically assigned
K_MN-*MAG-Auth_	a pre-shared authentication key between MAG and mobile node, where * stands for S: serving or C: candidate
MN-AAA-KEY	a shared key between mobile node AAA server
PBU	Proxy Binding Update
PBA	Proxy Binding Acknowledgement
HNP	Home Network Prefix
SMAG-ID	Serving MAG Unique identity of MAG
*E*….∥…. ∥ …_*x*	Concatenated and encrypted values under ‘x’key
hash()	a one way hash function
K*_MAG-LMA_	a pre-shared authentication key between MAG and LMA, where * stands for S: serving or C: candidate
HMAC	hash message authentication code
K*_MAG-AAA_	shared key between MAG and AAA server,where * stands for S: serving or C: candidate
SQN_SQN-index_	a sequence captioned by index of the sequence or order of sequence
KI_i_	*i*^*th*^ unique key identifier of master key K that would be re-assigned after each successful execution of handover protocol
LookUpTable_i_	*i*^*th*^ copy of random number lookup table
KDF	*i*^*th*^ Key Derivation Function(cipher key derivation function)
CxMAG-ID	Unique identity of Candidate MAG, where x is 1,2,3……..,n assuming there would be n number of candidate MAGs
K_MN-LMA_	shared key between MN and LMA

## 5 Security analysis

### 5.1 Resisting compromised MAG

During handover, a compromised serving MAG can cause denial of service by ignoring the presence of newly detected MN, redirecting to unknown entity pretending it is to LMA or de-registering existing MN from LMA entry table as it has handed-over to another mobility domain. For this threat to happen, MAG can ignore an attachment request of mobile node or it is when the MAG ignores a solicitation request from MN. In either of the cases, the MN waits a response (attachment success signal, router advertisement signal or traffic data) for predefined time interval. If the node does not receive any response with in predefined time interval or receives negative response from a serving MAG (*SMAG*), it indicates that the access router is compromised, failed or power turned off; then the MN sends a Handover control signal directly to LMA, digesting under key *K*_MN-LMA_ which would be shared during preceding handover with LMA.

As a result, the LMA starts data buffering and manages handover procedure with available candidate MAGs in the network mobility domain, in which security requirements are guaranteed or maintained, in the expense of local mobility protocol flow being out of order and handover delay. These proposed procedures can also be taken as a recovery procedures whenever a serving MAG fails to manage the handover process due to different circumstances. As a detail protocol flow of this solution shown is not shown explicitly in Figs 3 & 4 for such a scenario, it is summarized here below:

It is indicated in the protocol that the key is formed as:

$$K_{\text{MN-LMA}}=KDF\left(LookUpTable_{x+y},K_{\text{Channel}}\right),$$

where *KDF*() is predetermined Key Derivation Function, *x* is selected by MN among pre-generated random numbers and *y* is a constant agree on, in advance. The reset of procedural execution steps are illustrated as follows:

1. From the instance MN learns serving MAG is not responding to handover request for the predefined time interval, it sends a handover/recovery request to LMA along with encrypted new random number(*LookUpTable*_x′+y_) and key identifier(*KI*_i+1_) using *K*_i_ which was shared initially or during preceding handover with AAA server, where *x*′ is newly selected table index. Here, even-though an intruder may access access *x*′, the LookUpTable_x′_ will remain hidden to MN and AAA server because it is stored securely at either side only. The request would be compiled as:
  *Recovery-Request* ∥ *H*_1_ ∥ *E*(*KI*_i+1_ ∥ *x*′)_*K*_i_, where *H*_1_ = *HMAC*(*K*_MN-LMA_, *LMA-ID* ∥ *MN-ID*), *K*_i_ = *Crypto-Alg*-2(*LookUpTable*_x+y_, *MN-AAA-KEY*, *KI*_i_).
2. Receiving the handover/recovery request from MN, LMA validates the legitimacy of the mobile node by computing:
  $$HMAC\left(K_{\text{MN-LMA}},LMA-ID∥MN-ID_{i+1}\right)=H_{1}?$$
  Subsequently, LMA hands-over the request to AAA so that the authentication server creates new group key K_group_ with the remaining group members with in local mobility domain, considering the compromised MAG as a leaving member of the group. A procedure of formation of new group key would be in the same way with section 4.4, except *i*^*th*^ compromised MAG’s $g^{a_{\text{i}}}$ is to be excluded.
  $$K_{\text{group}}={\hat{e}}_{n-1}\left(g^{a_{1}},g^{a_{2}},...,g^{a_{n-1}}\right)={\hat{e}}_{n-1}{\left(g,g,...,g\right)}^{a_{1}a_{2}...a_{n-1}}.$$
3. The AAA server selects a new number from group sequence number list (SQN_SQN-index’_), where SQN-index’ is newly selected index of predetermined number sequence. Encrypting under the new group key *K*_group_, the server sends the selected index SQN-index’ and new mobile *MN-ID*_i+1_. i.e *E*(*MN-ID*_i+1_ ∥ *SQN-index*′ ∥ x’)_*K*_group_.
4. Once the LMA receives the message, it sends a resource query *MIH-N*2*N-HO-Query-Resources-request* ∥ *E*(*MN-ID*_i+1_ ∥ *SQN-index*′)_*K*_group_ ∥ *H*2) to candidate MAGs, where *H*2 = *HMAC*(*K*_group_, *SMAG-ID* ∥ *SQN*_SQN-index’_)
5. Verifying authentication message *H*2, the candidate MAGs respond to LMA with resource availability information including their authentication code in the form of:
  $$MIH-N2N-HO-Query-Resources-response∥H3=HMAC(K_{\text{group}}∥C*MAG∥SQN_{\text{SQN-index'}}),$$
  where * stands for numbers 1,2,3…..m for m number of candidate MAGs.
6. Finally, the LMA sends MIH-N2N-HO- Query-Commit- request for which the selected MAG responds with MIH-N2N-HO- Query-Commit- response and the remaining handover process is the same with section 4.4.1.

### 5.2 Resisting compromised LMA

Once LMA is compromised, it can redirect all packets or handover request signals to a single MAG, which results in denial of service. It may also ignore the handover request or IP tunneled packets from and/or to the mobile node. In our MIH assisted PMIPv6 mobility domain security protocol,redirecting MN handover request to a single MAG may be attempted when LMA may trick in to believing the mobile node that the MAG to handover to is really the one which sent MIH-N2N- HO-Query-Commit-response to the serving MAG. There is no any involvement for LMA in L2(MIH) handover execution procedures to manipulate message flow or identity of selected MAG when our proposed scheme is concerned. Beside that, at the beginning of L3 handover process, the selected MAG sends is hashed identity under preshared key *K*_C1MAG-AAA_ to AAA server. This will be verified. i.e. *HMAC*(*K*_C1MAG-AAA_, *C*1*MAG-ID* ∥ *M*8) shall be verified by AAA server and MN at step l and m of section 4.4.1. As a result, a compromised LMA can not successfully trick MNs to redirect to a single non-selected MAG.

On the other hand, because there is no need for the LMA to involve in the L2 MIH message flow processes, there is no possibility to ignore handover request from MN.

### 5.3 Resistant to mobile node impersonation

An attacker that is able to forge an identity of a mobile node can trick a MAG into believing there is a handover request from a legitimate user or redirecting data plane packets to attacker. In the proposed scheme a mobile identity *MN − ID*_i_ is dynamically set and sent to the network by encrypting or hashing under shared keys during handover process. The mobile identity is possibly sent onto communication channels, ultimately to AAA server, in two cases in our handover scheme, i.e., during L2 attachment and router solicitation requests. In both cases, it is hashed as message authentication code for validity verification using a key *MN-AAA-KEY* which itself is derived from master key *K* obtained during initial authentication and encrypted under *i*^*th*^ key *K*_i_ that is generated as *Crypto-Alg*-2(*LookUpTable*_x+y_, *MN-AAA-KEY*, *KI*_i_) in case of L2 attachment request, where *x* and *y* are pre-generated pseudo-random number index and pre-defined constant. This guarantees a secrecy of *MN-ID* and there is no loophole for an attacker to copy fraudulently.

### 5.4 Resisting man-in-the-middle attack

An attacker that can interpose between a mobile node and a MAG during link- and/or IP-layer Hanover signaling, may be able to launch a man in-the-middle attack on the mobile node by tricking the mobile node into believing that it has a legitimate connection with the localized mobility management domain, which enables the attacker to intercept, inspect, modify, or drop data plane packets from or to the mobile node. During the proposed handover protocol execution there are only two messages communicated between MN and MAGs. One is to send a handover/L2 attachment request to serving MAG and the other is a query of router solicitation sent to selected candidate MAG. While requesting L2 attachment, the MN sends its and MAG’s digested identity to serving as MN’s identity is unique and dynamically fulfills freshness needs not to replay. MAG verifies this as shown bellow: *HMAC*(*K*_MN-SMAG-Auth_, *SMAG-ID* ∥ *MN-ID*_i_) = *M*_auth_?

On the other hand, when the mobile node sends a router solicitation to the selected MAG, a new *MN-ID*_i+1_ is set and hashed using newly generated key *K*_MN-C1MAG-Auth_ = *Crypto-Alg*-1(*LookUpTable*_x+y_, *MN-AAA-KEY*, *KI*_i+1_).

The message that would be sent to the MAG and then relayed to AAA server for authentication is *Router-Solicitation* ∥ *M*8 = *HMAC*(*MN-AAA-KEY*, (*LookUpTable*_x+y_ ∥ *MN-ID*_i+1_) ⊕ *KI*_i+1_).

Here, the server authenticates the MN on *M*8 and the MAG on authentication message *M*10 (see section 4.4.1).

As a response to solicitation request, an acknowledgment shall be sent to MN which includes authentication code, *HMAC*(*K*_MN-C1MAG-Auth_, *MN-ID*_i+1_ ∥ *C*1*MAG-ID*) as accreditation validation for MN.

For the man in-the-middle attacker to trick in to believing it is a legitimate, it should be able to generate these messages and obtain data plane security keys, which is practically undo-able or not polynomial time task.

### 5.5 Mitigating replay attack

An adversary can resend a handover request message sent earlier from legitimate user, in order to impersonate the MN and some other entities with in the local mobility network. The proposed scheme can mitigate this, as there are security credentials generated dynamically or newly assigned (fresh) whenever handover request is triggered. For the handover messages between authentication server and the MN, *MN-ID*_i_ is dynamically generated; where as *LookUpTable*_x_, key identifier *KI*_i_ and shared key *K*_i_ are fresh for each complete handover process. In case of messages exchanged between MN and MAGs, authentication keys *K*_MN-C*MAG-Auth_, *K*_MN-SMAG-Auth_, and *K*_MN-LMA_ are newly generated so that replay attack can be resisted.

### 5.6 Verifier impersonation

This kind of impersonation is one of the scenarios for impersonation to happen. This is said to be happened when adversary creates independent connection with the victims and exchanges messages with, causing them to think that they are directly talking to each other. In our proposed secure handover protocol, to be successful with that, the adversary should at least be able to generated mutual authentication messages exchanged between MN and MAGs. MN authenticates a MAG it hands-over to on *HMAC*(*K*_MN-C1MAG-Auth_, *MN-ID*_i+1_ ∥ *C*1*MAG-ID*) and the MAG authenticates the MN on *HMAC*(*K*_MN-C1MAG-Auth_, *MN-ID*_i+1)_. In addition to that, IDs of the MN and the MAG, which can be IP address of these parties, are included within authentication messages so that data plane IP packets will be tunneled by. The attacker has to get authenticated by MN as a MAG and has to own the same ID with that digested within authentication message in order it is said to be successful in Verifier Impersonation. Moreover, for communications among member groups(MAGs, LMA and AAA server), there is a fresh sequence of number(*SQN*_S_*QN-index*) selected each time handover is triggered. As a result, the scheme is definitely considered as a resistant to the this threat.

### 5.7 Server stolen-verifier

Storing MN’s verification table may result in stolen-verifier attacks. In our proposed protocol, security credentials stored at authentication server and MN side are random number table *LookUpTable*_x_, key identifier *KI*_i_. An intruder may steal these parameters and then may forge an identity of the mobile node. But, the security keys which are derived from a security stored master key *K* have to be obtained for the intruder to be successful in forging MN’s identity as mobile node identity *MN-ID*_i_ is generated from *LookUpTable*_x_, *KI*_i_ and the derived keys.

### 5.8 Location privacy

Over decades, location privacy has been a big concern for mobile users as it could be tracked easily. Exposing MN’s identity is one of the reasons for mobile node not to have Location Privacy. The proposed scheme mitigates this through assigning a identity of the MN dynamically and sending over network securely. Except the authentication server and MN itself, there is no entity which the mobile identity may be exposed to. This makes the scheme a solution to guarantee Location Privacy.

Keeping the MN’s IP address or Mobile node identity fixed and easily accessible over the PMIPv6 domain, during initial connection attachment or when executing handover procedure, may result in location privacy policy violation.

## 6 Formal security analysis

### 6.1 BAN logic analysis

BAN Logic is a well-known authentication logic created to assist a validation of authentication protocols which remains popular with many protocol designers. It is a set of rules for defining and analyzing knowledge & beliefs of involved parties in authentication protocol in a formal manner. Specifically, BAN logic helps security protocol designers to determine whether exchanged information is trustworthy, secured against eavesdropping, or both. Generally, an analysis based on BAN logic comprises three steps: (i) idealizing message flow of a protocol (ii) determining protocol assumptions and setting goals (iii) deriving beliefs with respect to the rules and proving the defined goals.

Before going in to details of logic analysis steps of ban logic, let’s briefly summarize a set rules and symbolic notations as follows:

- Notations:
- Message-meaning rule:
$\frac{P|\equiv P\overset{K}{↔}Q,P⊲{\left\{X\right\}}_{\text{K}}}{P|\equiv Q|\sim X}$ : If P believes that it shares a a secret key K with Q, and if P receives a message containing X encrypted with K then P believes that Q once said X(see Table 2 for symbol notation description).
- Nonce-Verification Rule:
$\frac{P|\equiv \#(X),P|\equiv Q|\sim X}{P|\equiv Q|\equiv X}$ : if P believes that X is a fresh message, and P believes that it was said by Q than P believes that Q believed once believed X.
- Jurisdiction Rule:
$\frac{P|\equiv Q\Rightarrow X,P|\equiv Q|\equiv X}{P|\equiv X}$ : if P believes that the principal Q jurisdiction has a control over X and if P believes that Q believes it to be true, then P must believe in it also.
- Session Key Rule:
$\frac{P|\equiv \#(X),P|\equiv Q|\equiv X}{P|\equiv P\overset{K}{↔}Q}$ : If the entity P believes that X is fresh and Q believes X, then P believes the secret K that is shared between both entities P and Q.
- Freshness-conjuncatenation Rule:
$\frac{P|\equiv \#(X)}{P|\equiv \#(X,Y)}$ : If the entity P believes that X is fresh, then P believes the freshness of (X, Y).
- Believe Rule 1:
$\frac{P|\equiv X,P|\equiv Y}{P|\equiv (X,Y)}$ , if p believes X and P believes Y, then P believes a collection (X,Y).
- Believe Rule 2:
$\frac{P|\equiv (X,Y)}{P|\equiv X,P|\equiv Y}$ , if p believes X and Y collectively, then P believes a X and Y individually.

**Table 2:** BAN logic notations and respective descriptions.

Notations	Descriptions
*P*∣≡*X*	An entity *P* believes a statement *X*
*P* ⇒ *X*	*P* has jurisdiction on a statement *X*
*P*∣∼*X*	*P* once said *X*
*P*⊲*X*	*P* sees *X*
{*X*}_*K*_	*X* in encrypted under key *K*
$P\overset{K}{↔}Q$	*P*and *Q* share key *K*
*P* → *Q*: m	*P* sends a message m and *Q* receives it
#(*X*)	*X* is fresh

i Idealizing the Message Flow:
  Initially, the message of handover authentication processes for the proposed scheme would be idealized as follows:
  **(M_1_)**  *MN* → *SMAG*:
     {L2-HO-Triggering, (x:TableIndex, MN-ID_i_)K_i_, (LookUpTable_x_, MN-ID_i_, KI_i_)_MN-AAA-KEY_, (OTP_i_, MN-ID_i_)K_MN-SMAG-Auth_}
  **(M_2_)**  *SMAG* → *AAA*:
     {(TableIndex, MN-ID_i_)K_i_, (SMAG-ID, (LookUpTable_x_, MN-ID_i_, KI_i_)_MN-AAA-KEY_)K_SMAG-AAA_}
  **(M_3_)**  *AAA* → *SMAG*:
     {(SMAG-ID, SQN-index)K_group_ }
  **(M_4_)**  *SMAG* → C*MAG:
     {MIH-N2N-HO-Query-Resources-request, (SMAG-ID, SQN-index)K_group_}
  **(M_5_)**  C*MAG → *SMAG*:
     {MIH-N2N-HO-Query-Resources-response, (C*MAG-ID, SQN_SQN-index_)K_group_}
  **(M_6_)**  *MN* → *C*1*MAG*:
     {Router-Solicitation,(LookUpTable_x+y_, MN-ID_i+1_, KI_i+1_)_MN-AAA-KEY_, (MN-ID_i+1_)K_MN-C1MAG-Auth_}
  **(M_7_)**  *C*1*MAG* → *LMA*:
     {(C1MAG-ID, (LookUpTable_x+y_, MN-ID_i+1_, KI_i+1_)_MN-AAA-KEY_, (LMA-ID, PBU)K_C1MAG-AAA_}
  **(M_8_)**  *LMA* → *AAA*:
     {(C1MAG-ID, (LookUpTable_x+y_, MN-ID_i+1_, KI_i+1_)_MN-AAA-KEY_, (LMA-ID, PBU)K_C1MAG-AAA_, (LMA-ID)K_LMA-AAA_}
  **(M_9_)**  *AAA* → *LMA*:
     {(LMA-ID, K_MN-C1MAG-Auth_, MN-ID_i+1_)K_C1MAG-AAA_}
  **(M_10_)**  *LMA* → *C*1*MAG*:
     {(LMA-ID, HNP)K_MN-LMA_, (HNP, LMA, ID)K_MN-LMA_, PBA, (K_MN-C1MAG-Auth_, MN-ID_i+1_)K_C1MAG-AAA_}
  **(M_11_)**  *C*1*MAG* → *MN*:
     {Router-Advertisement, (LMA-ID, HNP)K_MN-LMA_, (HNP, LMA, ID)K_MN-LMA_, (MN-ID_i+1_, C1MAG-ID)K_MN-C1MAG-Auth_}
ii Assumptions:
  Initial assumptions, from which goals are derived, have to be set to prove the security of the proposed scheme using BAN logic as listed below:
  **(A_1_)** $MN|\equiv \left(MN\overset{K_{\text{i}}=H(LookUpTable_{\text{x}},MN\text{-}AAA\text{-}KEY,KI_{\text{i}})}{↔}AAA\right)$
  **(A_2_)** $AAA|\equiv \left(MN\overset{K_{\text{i}}=H(LookUpTable_{\text{x}},MN\text{-}AAA\text{-}KEY,KI_{\text{i}})}{↔}AAA\right)$
  **(A_3_)** $SMAG\left(\overset{K_{\text{MN-SMAG-Auth}}=H(LookUpTable_{\text{x}},MN\text{-}AAA\text{-}KEY,KI_{\text{i}})}{↔}\right)MN$
  **(A_4_)** $C*MAG\left(\overset{K_{\text{MN-C}*\text{MAG-Auth}}=H(LookUpTable_{\text{x}+\text{y}},MN\text{-}AAA\text{-}KEY,KI_{\text{i}+1})}{↔}\right)MN$
  **(A_5_)** $MN|\equiv \#(LookUpTable_{\text{x}})$
  **(A_6_)** $AAA|\equiv \#(LookUpTable_{\text{x}})$
  **(A_7_)** $MN|\equiv \#(KI_{\text{i}})$
  **(A_8_)** $AAA|\equiv \#(KI_{\text{i}})$
  **(A_9_)** $MN\left(\overset{MN\text{-}ID_{\text{i}}=hash(LookUpTable_{\text{x}}⊕MN\text{-}ID_{\text{i}}⊕MN\text{-}AAA\text{0}KEY)}{↔}\right)AAA$
  **(A_10_)** $SMAG|\equiv SMAG\overset{K_{\text{SMAG-AAA}}}{↔}AAA$
  **(A_11_)** $AAA|\equiv SMAG\overset{K_{\text{SMAG-AAA}}}{↔}AAA$
  **(A_12_)** $AAA|\equiv C*MAG\overset{K_{\text{C}*\text{MAG-AAA}}}{↔}AAA$
  **(A_13_)** $C*MAG|\equiv C*MAG\overset{K_{{}_{\text{C}1\text{MAG-AAA}}}}{↔}AAA$
  **(A_14_)** $SMAG|\equiv SMAG\overset{K_{\text{group}}}{↔}AAA$
  **(A_15_)** $AAA|\equiv SMAG\overset{K_{\text{group}}}{↔}AAA$
  **(A_16_)** $AAA|\equiv C1MAG\overset{K_{\text{group}}}{↔}AAA$
  **(A_17_)** $C*MAG|\equiv C1MAG\overset{K_{\text{group}}}{↔}AAA$
  **(A_18_)** $SMAG|\equiv \#\left(OTP_{\text{i}}\right)$
  **(A_19_)** $C*MAG|\equiv \#(OTP_{\text{i}+1})$
  **(A_20_)** $SMAG\equiv \#(MN\text{-}ID_{\text{i}})$
  **(A_21_)** $C*MAG|\equiv \#(MN\text{-}ID_{\text{i}+1})$
  **(A_22_)** $MN|\equiv \#(MN\text{-}ID_{\text{i}+1})$
  **(A_23_)** $MN\equiv \#(MN\text{-}ID_{\text{i}})$
  **(A_24_)** $AAA|\equiv \#(MN\text{-}ID_{\text{i}})$
iii Goals:
  The proposed handover scheme should satisfy the following goals to prove its security under BAN logic, applying the assumptions and the set of rules defined.
  **(G_1_)** $SMAG|\equiv SMAG\overset{K_{\text{MN}-\text{SMAG-Auth}}}{↔}MN$
  **(G_2_)** $C*MAG|\equiv C*MAG\overset{K_{\text{MN}-{\text{C}}^{*}\text{MAG-Auth}}}{↔}MN$
  **(G_3_)** $MN|\equiv C*MAG\overset{K_{\text{MN}-{\text{C}}^{*}\text{MAG-Auth}}}{↔}MN$
  **(G_4_)** $AAA|\equiv AAA\overset{MN-AAA-KEY}{↔}MN$
  **(G_5_)** *AAA* ∣≡ *MN* ∣≡ *MN-ID*_i+1_
iv Proofs:
  Now, utilizing BAN-Logic postulates rules and the assumptions, we prove or derive set goals.
  **V1**: From message M1, we know that
    $$SMAG⊲{\left(OTP_{\text{i}},MN-ID_{\text{i}}\right)}_{{\text{K}}_{\text{MN-SMAG-Auth}}}$$
  **V2**: Having V1, A3 and referring message-meaning rule:
    $$SMAG|\equiv MN|\sim \left(OTP_{\text{i}},MN-ID_{\text{i}}\right)$$
  **V3**: From A18, A20 and Freshness rule:
    $$SMAG|\equiv \#\left(OTP_{\text{i}},MN-ID_{\text{i}}\right)$$
  **V4**: From V2, V3, and Nonce-verification rule:
    $$SMAG|\equiv MN|\equiv \left(OTP_{\text{i}},MN-ID_{\text{i}}\right)$$
  **V5**: Accordingly, based on V3, V4 and Session key rule, **G1** will be proved as:
    $$SMAG|\equiv SMAG\overset{K_{\text{MN}-\text{SMAG-Auth}}}{↔}MN$$
  **V6**: From massage M6, we know that
    $$C*MAG⊲{\left(MN-ID_{\text{i}+1}\right)}_{{\text{K}}_{\text{MN}-{\text{C}}^{*}\text{MAG}-\text{Auth}}}$$
  **V7**: By taking the assumption A4, V6 and the message-meaning:
    $$C*MAG|\equiv MN|\sim \left(MN-ID_{\text{i}+1}\right)$$
  **V8**: based on V7, A21 and Nonce-verification rule:
    $$C*MAG|\equiv MN|\equiv \left(MN-ID_{\text{i}+1}\right)$$
  **V9**: the second goal(**G2**) can be derived from V8 on A21:
    $$C*MAG|\equiv C*MAG\overset{K_{\text{MN}-{\text{C}}^{*}\text{MAG}-\text{Auth}}}{↔}MN$$
  **V10**: From massage M11, one can deduce that:
    $$MN⊲{\left(MN-ID_{\text{i}+1},C*MAG-ID\right)}_{{\text{K}}_{\text{MN}-{\text{C}}^{*}\text{MAG-Auth}}}$$
  **V11**: By taking the assumption A4, V10 and the message-meaning rule:
    $$C1MAG|\equiv MN|\sim (MN\text{-}ID_{\text{i+1}},C*MAG\text{-}ID)$$
  **V12**: Having the assumption A22, and Freshness rule:
    $$MN|\equiv \#(MN\text{-}ID_{\text{i+1}},C*MAG\text{-}ID)$$
  **V13**: Considering V11, V12 and and Nonce-verification rule:
    $$MN|\equiv C*MAG|\equiv (MN\text{-}ID_{\text{i+1}},C*MAG\text{-}ID)$$
  **V13**: then **G3** shall be proved:
    $$MN|\equiv C*MAG\overset{K_{\text{MN-C}*\text{MAG-Auth}}}{↔}MN$$
  **V14**: From message M1:
    $$MN⊲({\left(x:TableIndex,MN\text{-}ID_{\text{i}}\right)}_{{\text{K}}_{\text{i}}})$$
  **V15**: Referring assumption A1, V14 and the message-meaning rule:
    $$AAA|\equiv MN|\sim (x:TableIndex,MN\text{-}ID_{\text{i}+1})$$
  **V16**: From A5, A23, V15 and Nonce-verification rule:
    $$AAA|\equiv MN|\equiv (MN\text{-}ID_{\text{i}})$$
  **V17**: as a result, using Session key rule:
    $$AAA|\equiv AAA\overset{MN\text{-}AAA\text{-}KEY}{↔}MN,$$
    which is **G4**
  **V18**: From message M8:
    $$MN⊲({\left(LookUptable_{\text{x+y}},MN\text{-}ID_{\text{i+1}},KI_{\text{i+1}}\right)}_{\text{MN-AAA-KEY}})$$
  **V19**: Referring assumption V17, V18 and the message-meaning rule:
    $$AAA|\equiv MN|\sim ((LookUptable_{\text{x+y}},MN\text{-}ID_{\text{i+1}},KI_{\text{i+1}})$$
  **V20**: From A24, V19 and Nonce-verification rule **G5** is achieved that:
    $$AAA|\equiv MN|\equiv (MN\text{-}ID_{\text{i+1}}).$$

### 6.2 Analysis under avispa

To illustrate the validity and security capability of the proposed protocol, the vulnerability of the protocol is strongly analysed under Automated Validation of Internet Protocols and Applications (AVISPA)tool. The protocol specification has five participants that are represented by five basic roles. These are mobile node (MN), Local Mobility Anchor (LMA), Serving Mobility Access Gateway (SMAG), Candidate Mobility Access Gateway (CMAG) and AAA server, where these entities are denoted as mn, router1, router2, server and lma respectively in the protocol simulation. Freshness, Secrecy and authenticity of of *MN-ID*_i_, *LookUpTable*_x_, *KI*_i_, *OTP*_i_, and *SQN*_SQN-idex_ are some of the defined goals. The server authenticates, MN, MAG as well as LMA on these security credentials and shared keys. MN and MAGs are also mutually authenticated each other on their Unique identities and their shared keys. These all together are set as analysis goals.

**Fig 5:**
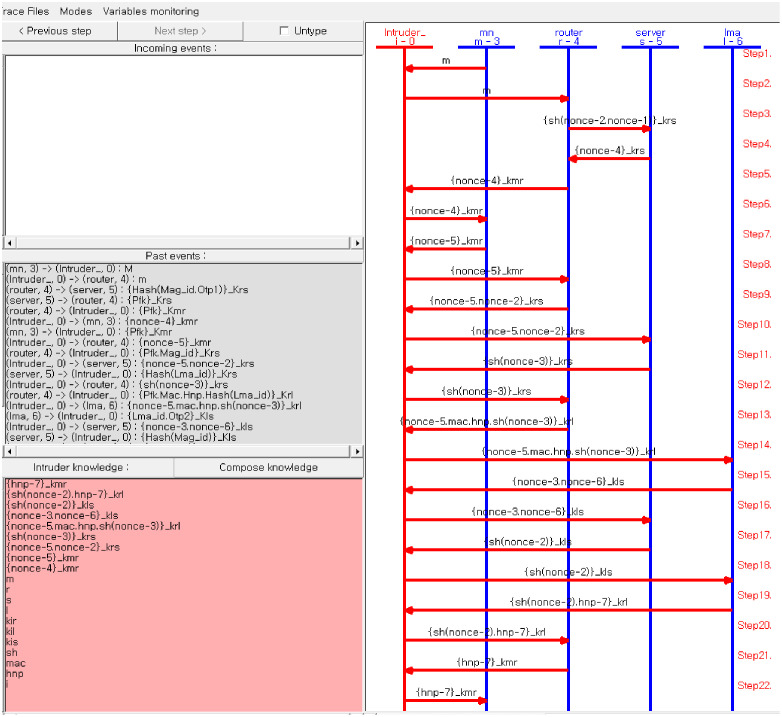
Protocol execution simulation.

Unless animated, there is no guarantee that the message sent from the participants reaches the destination or received by the receiver entity. One can obtain all interleaving between MN, MAG, AAA server and LMA by animating the protocol on SPAN, which is edition and animation tool of AVISPA high-level security protocols verification environment. It also enables to trace the values of the variables whether the correct values are being exchanged within the simulated entities. Hence, the Authors prefer to show the procedural execution of source specification of the newly proposed protocol as it is illustrated in Fig 5. Depending on its pre-defined knowledge, an intruder may try to authenticate the mobile to itself by forging the authentication massages intended for another entity because the intruder is the network.

**Fig 6:**
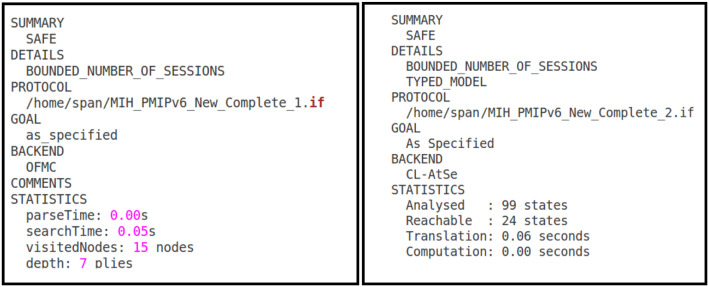
Avispa verification result.

Under all defined circumstances within the protocol specification, the proposed protocol is found to be SAFE in all back-ends of AVISPA against all possible attacks as shown in Fig 6.

In GOAL section of the simulation result, it is reported that the goals are satisfied as specified. In other words, the intended security services are achieved. From specified model, one can retrieve modes of message and there is the possibility to check the values of the variables of each participant. The user chooses the variables of each role he wants to monitor. Accordingly, the author followed these procedures to validate the protocol and in general, strong authentication is considered and verified under aforementioned formal analysis session.

## 7 Performance analysis

Under this section, performance analysis of the proposed solutions will be illustrated in terms of signaling cost and handover latency as a performance metric. We compare our solution with the FPMIPv6 handover standard protocol [13] and MIH_−_SPFP [43]using the performance metric.

We analyzed the performance metric by varying subnet radius, wireless link failure probability, the speed of the mobile, number of hops, wireless link layer frame error rate and other parameters, where the network model is assumed to be LTE network.

### 7.1 Handover delay analysis

The handover delay is the time interval between the moments when an MN loses connectivity with its serving MAG (SMAG) until the moment it receives the first packets from the candidate MAG (C*MAG). To analyze handover delay and Signaling Cost of the protocols for comparisons, we adapt the method in [29].

Suppose *τ* is inter-frame time, *ρf* is the frame error rate over the wireless link, where as *Lp* and *Lf* are the packet size (typically an IP packet) and frame size respectively, and *D*_wl_ is the wireless link delay.

Assume *p*_i,j_ is probability that the first frame sent from the MN arrived at Access Router (AR/MAG) successfully, which is *i*^*th*^ re-transmitted frame at the *j*^*th*^ re-transmission trial. Having this, a one-way frame transportation delay *d*_frame_ between the MN and the AR through the wireless link can be expressed as:

$$\begin{array}{c} d_{\text{frame}}=D_{\text{wl}}\left(1-\rho f\right)+\sum_{i=1}^{n}(\sum_{j=1}^{i}p_{i,j}\left(2i*D_{\text{wl}}+2\left(j-1\right)\tau \right) \end{array}$$ (1)

where *i* ≤ *n*, *j* ≥ *i*, and *D*_wl_ is mainly depending on L2 technology being utilized.

$$\begin{array}{c} p_{i,j}=\rho f{\left(1-\rho f\right)}^{2}{\left(\left(2-\rho f\right)\rho f\right)}^{\left(\left(i^{2}-i\right)2+j-1\right)} \end{array}$$ (2)

Assuming *k* is the number of frames per packet over the wireless link, it can be expressed as:

$$\begin{array}{c} k=\frac{L_{p}}{L_{f}} \end{array}$$ (3)

where *L*_p_ and *L*_f_ are the packet size and the frame size, respectively.

From (1)–(3):

$$\begin{array}{c} d_{\text{wl}}\left(Lp\right)=d_{\text{frame}}+\left(k-1\right)\tau \end{array}$$ (4)

Assuming there is no packet loss in case of wired links and will reach the destination without re-transmission, the one way packet transportation delay over the wired link *d*_wd_(*Lp*) can be obtained as follows:

$$\begin{array}{c} d_{\text{wd}}\left(Lp\right)=\frac{L_{P}}{BW_{\text{wired}}}+D_{\text{wired}} \end{array}$$ (5)

where *BW*_wired_ and *D*_wired_ are the bandwidth and the latency of wired links, respectively. A one-way packet transportation delay over a wired link through h hops can be expressed as:

$$\begin{array}{c} d_{\text{wd}}\left(Lp,h\right)=\frac{L_{P}*h}{BW_{\text{wired}}}+D_{\text{wired}} \end{array}$$ (6)

From [29] we learn that handover delay of Fast proxiy mobile IP version six (FPMIPv6) handover scheme is expressed as:

$$\begin{array}{c} t_{\text{FH}}=T_{L_{2}}+d_{\text{wl}}\left(M_{1}\right)+d_{\text{wl}}\left(L_{D}+40\right) \end{array}$$ (7)

As one of the selected schemes for comparison, MIH-SPFP [43] handover delay is defined as follows:

$$\begin{array}{c} t_{\text{MIH-SPFP}}=T_{L_{2}}+d_{\text{wl}}\left(M_{2}\right)+d_{\text{wl}}\left(L_{D}+40\right)+d_{\text{wl}}(M_{\text{Auth-WL}}+d_{\text{wd}}(M_{\text{Auth-Wired}}), \end{array}$$ (8)

where *d*_wl_(*M*_Auth-WL_ and *d*_wd_(*M*_Auth-Wired_ are time delays due to authentication messages ove MIH-SPFP scheme over wirelesses and wired links respectively.

A handover delay of the proposed scheme incurs an additional time delay of *t*_Auth_ as compared to FPMIPv6, where *t*_Auth_ is a time required to complete the proposed handover authentication and security credentials processing. Assuming we are implementing HMAC SHA256 for all message authentication codes of our protocol and processing time is negligible, *t*_Auth_ would be determined as:

$$\begin{array}{c} t_{\text{Auth}}=D_{\text{wl}}\left(M_{\text{Pro-Auth-WL}}\right)+D_{\text{wd}}(M_{\text{Pro-Auth-Wired}}), \end{array}$$ (9)

where *D*_wl_(*M*_Pro-Auth-WL_) and *D*_wd_(*M*_Pro-Auth-Wired_) are time delays due to authentication messages of proposed scheme over wirelesses and wired links respectively.

$$t_{\text{Proposed}}=T_{{\text{L}}_{2}}+d_{\text{wl}}(M_{1})+d_{\text{wl}}(L_{\text{D}}+40)+D_{\text{wl}}(M_{\text{Pro-Auth-WL}})+D_{\text{wl}}(M_{\text{Pro-Auth-Wired}})$$ (10)

### 7.2 Signaling cost analysis

In performing signaling cost analysis, we adopted [29] model, which was a model for a city assumed to have a rectangular surface area (a*b) and mobile node moves in an epoch-based pattern across the city. Suppose *d*_x_ and *d*_y_ represent the distance between adjacent horizontal roads and vertical roads, respectively. From this, expected epoch length can be expressed as:

$$\begin{array}{c} E\left(L\right)=\frac{d_{x}\left(N_{h}+1\right)\left(N_{h}-1\right)}{3N_{h}}+\frac{d_{y}\left(N_{v}+1\right)\left(N_{v}-1\right)}{3N_{v}}, \end{array}$$ (11)

where *N*_h_ = *a*/*d*_x_ and *N*_v_ = *b*/*d*_y_.

Let *N*_x_ be the number of subnet crossings in an epoch for horizontal movements and *N*_y_ is for vertical movement. Number of expected subnet crossed by the mobile node can be evaluated as:

$$\begin{array}{c} E\left(N_{t}\right)=E\left(N_{x}\right)+E\left(N_{y}\right), \end{array}$$ (12)

where

$$\begin{array}{c} E\left(N_{x}\right)=\frac{m\left(m+1\right)K_{1}}{6{\left(N_{h}\right)}^{2}}\left(6N_{h}-4mK_{1}+K_{1}+3\right),E\left(N_{y}\right)=\frac{m\left(m+1\right)K_{2}}{6{\left(N_{v}\right)}^{2}}\left(6N_{v}-4mK_{2}+K_{2}+3\right) \end{array}$$ (13)

$$K_{1}=2r/d_{x},$$

where as *K*_2_ = 2*r*/*d*_y_ for which is r is a cell radius and there is at most m subnet crossing.

Suppose *V*_min_ and *V*_max_ are a minimum speed and maximum speed of mobile node respectively, expected time for an epoch can be defined as:

$$\begin{array}{c} E\left(T_{t}\right)=\frac{E\left(L\right)*ln|V_{\text{max}}/V_{\text{min}}|}{V_{\text{max}}-V_{\text{min}}} \end{array}$$ (14)

Assuming the mobile node movement to the destination is uniformly distributed over [0, *T*_(_ *max*)], the expected pause time *E*(*T*_p_) is calculated as 0.5*T_(_ *max*), where as the number of handover per unit time is expressed as:

$$\begin{array}{c} E(N_{c})=\frac{E(N_{t})}{E\left(T_{t}\right)+2E\left(T_{p}\right)} \end{array}$$ (15)

For wireless link transmission failing probability *P*_f_, signaling cost of the given solution can be calculated as:

$$\begin{array}{c} S=E\left(N_{c}\right)*P_{f}/\left(1-P_{f}\right)*S_{\text{FH-WL}}+S_{\text{FH-W}}), \end{array}$$ (16)

where *S*_FH-WL_ is the signaling cost of wireless link and *S*_FH-W_ is signaling cost of wired link.

Assuming the unit cost of on a wired link is *β* and wireless link is *α*, the signaling cost on wired and wireless links would be:

*S*_FH-WL_ = α*h_1_*M_wl_ and *S*_FH-W_ = β*h_2_*M_wired_, where *M*_wl_ is total communicated message over wireless link, *M*_wired_ is over wired link, *h*_1_ and *h*_2_ are average distances between nodes in the network.

Having the above equations, a signaling cost of fast PMIPv6 handover solution can be expressed as follows:

$$\begin{array}{c} S_{\text{FH}}=E\left(N_{c}\right)*\alpha [\frac{P_{f}}{1-P_{f}}H_{\text{MN-MAG}}\left(M_{1}+M_{2}+M_{7}+M_{8}+M_{19}+M_{23}+M_{24}\right)]\\ +E\left(N_{c}\right)*\beta [H_{\text{MAG-MAG}}\left(m\left(M_{11}+M_{12}\right)+M_{13}+M_{14}+M_{15}+M_{16}+M_{\text{HI}}+M_{\text{HAck}}\right)]\\ +E\left(N_{c}\right)*\beta [H_{\text{MAG-MIIS}}\left(M_{5}+M_{6}\right)+3H_{\text{MAG-LMA}}\left(M_{3}+M_{4}\right)] \end{array}$$ (17)

The signaling cost of MIH-SPFP solution is shown as follows:

$$\begin{array}{c} S_{\text{OH}}=E\left(N_{c}\right)*\alpha [\frac{P_{f}}{1-P_{f}}H_{\text{MN-MAG}}\left(M_{1}+M_{2}+M_{7}+M_{8}+M_{19}+M_{20}+M_{21}+M_{23}+M_{24}\right)]\\ +E\left(N_{c}\right)*\beta [H_{\text{MAG-MAG}}\left(m\left(M_{11}+M_{12}\right)+M_{13e}+M_{14e}+M_{15}+M_{16}+M_{22}\right)]\\ +E\left(N_{c}\right)*\beta [H_{\text{MAG-MIIS}}\left(M_{5}+M_{6}\right)+2H_{\text{MAG-LMA}}\left(M_{3}+M_{4}\right)] \end{array}$$ (18)

Similarly, we can calculate signaling cost of our proposed handover solution as follows, which would competitively be demonstrated numerically later.

$$\begin{array}{c} S_{\text{PROPOSED}}=E\left(N_{c}\right)*\alpha [\frac{P_{f}}{1-P_{f}}H_{\text{MN-MAG}}\left(M_{1}+M_{2}+M_{20}+M_{25}+M_{27}\right)]\\ +E\left(N_{c}\right)*\beta [H_{\text{MAG-MAG}}\left(m\left(M_{11}+M_{12}\right)+M_{13}+M_{23}+M_{24}+M_{15}+M_{16}++M_{26}\right)]\\ +E\left(N_{c}\right)*\beta [H_{\text{MAG-MIIS}}\left(M_{5}+M_{6}\right)+2H_{\text{MAG-LMA}}\left(M_{3}+M_{4}\right)] \end{array}$$ (19)

## 8 Numerical results and comparison

Here after, performance evaluation results of standard FPMIPv6, MIH-SPFP, and our proposed protocol shall numerically be illustrated in terms of handover delay and signaling cost.

### 8.1 Handover delay analysis results

For the numerical calculations, we assumed wireless link layer frame error rate (*ρf*) varies from 0 to 0.6, *L*_P_ = 1500 bytes which is a typical IPv6 packet size, *L*_F_ = 127 bytes and user data *L*_D_ is known to be 120 bytes. It is also noted that the message authentication code functions through out the comparative schemes are supposed to be HMAC-SH256, wired link bandwidth (*B*_wired_) is 10MHz, and delay over wired link (*D*_wired_) is 35msec.

**Fig 7:**
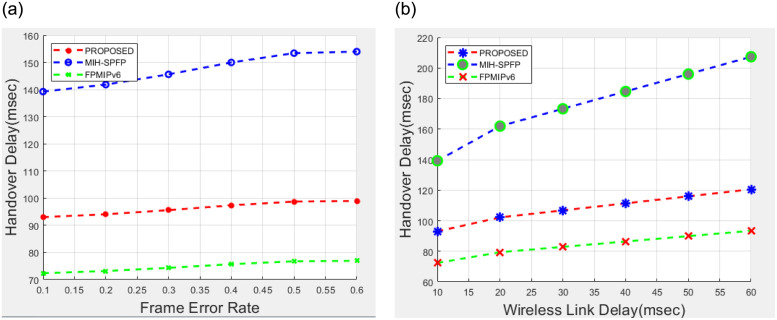
Handover delay comparison.

Fig 7(a) & 7(b) shows the handover latency as *ρp* varies and when wireless link delay differs. The higher *ρp* value, the higher handover delay due to re-transmission of packets, which is observed to be true for all the schemes under comparison. As shown in the Fig 7(b), the handover delay increases with wireless link delay as well. Comparatively, the standard FPMIPv6 without security solution has a better handover delay in the expense of fundamental security gaps as a trade off. Providing a security solution for the mobility management security threats, our proposed scheme is nearly selective as standard FPMIPv6. As discussed earlier, in our proposed protocol including L2 handover (MIH) and authentication procedures are fully managed by the network on the behalf of the mobile node. This decreases the wireless link delay and frame error rate significantly, which ultimately reduces over all handover delay.

### 8.2 Signaling cost analysis results

**Fig 8:**
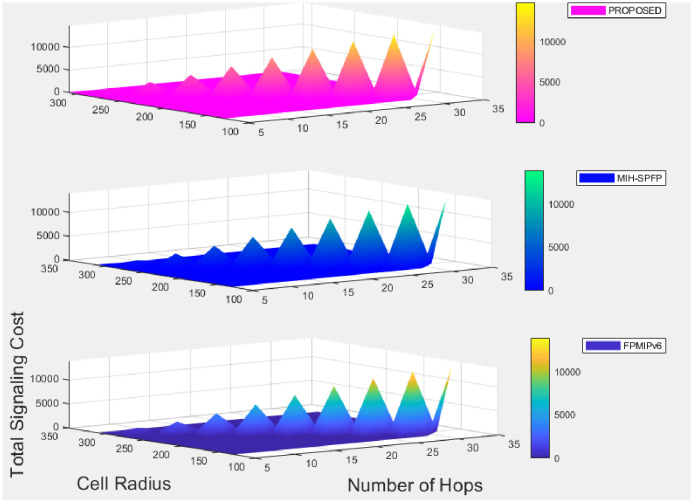
Wireless and wired total signaling cost comparison.

**Fig 9:**
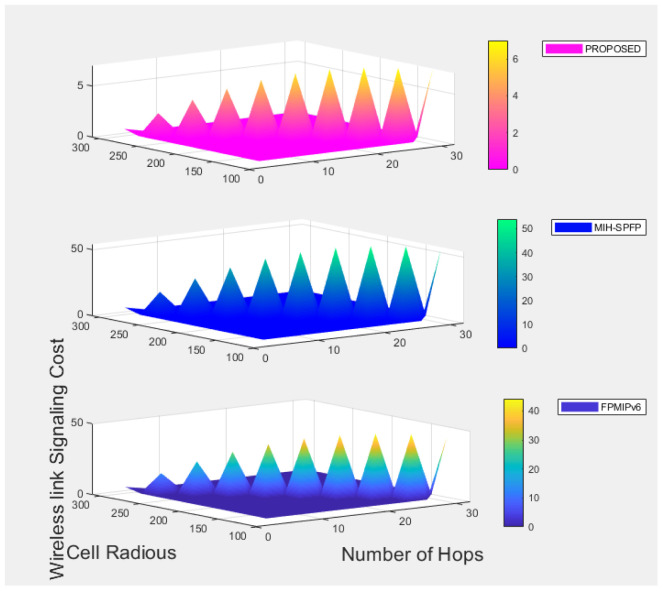
Wireless link signaling cost comparison.

**Fig 10:**
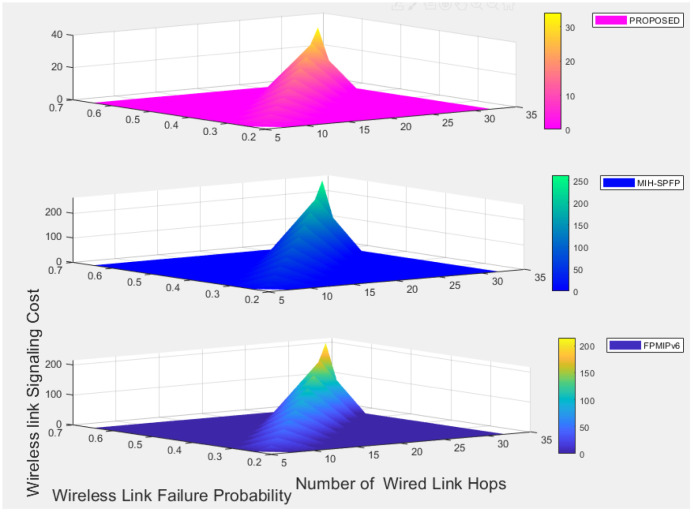
Wireless failure probability vs wireless link signaling cost.

**Fig 11:**
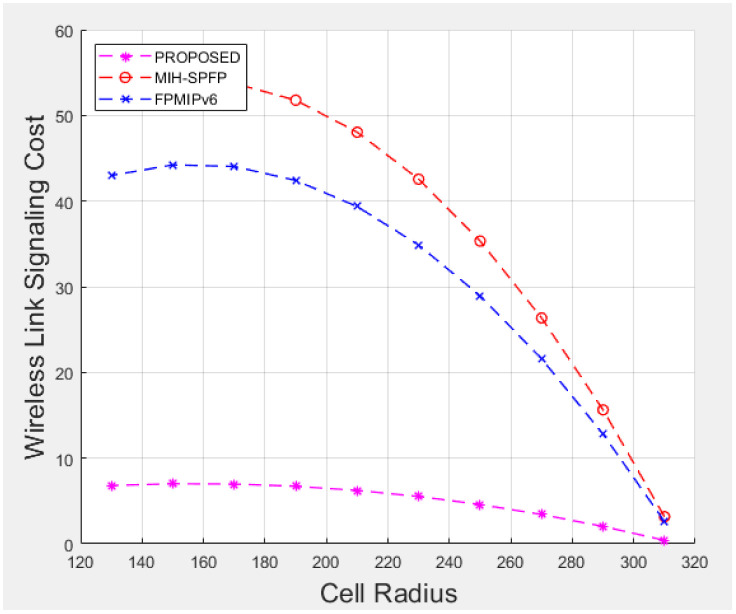
Wireless failure probability vs wireless link signaling cost.

Here below, applying mathematical equations under section 7.2, a signaling cost for each handover protocol is illustrated numerically having parameters and assumptions listed in Table 3 above, for which we referred most of the values from MIH-SPFP [43]. An average speed of the mobile node is assumed to vary from 1–50*m*/*s*, where as cell radius *r* of a subnet network is considered to be 130m–310m. Number of hops among all entities in wired link is supposed to be 5–32, maximum number of subnet crossed(*m*) = 15, and probability of failure (*ρ*) is changed from 0.2 to 0.65. In addition, a wireless signaling unit cost *β* = 2, *β* = 1.5, and for the sake of security, each time MN reaches a road intersection, it has to take a pause for a random duration between 0 and *T*_max_ of which its maximum is assumed to be 100sec. Referring parameters in Table 3, we evaluated the performance of our proposed protocol in comparison with the selected schemes as shown in Figs 8–11. Fig 8 shows the handover over all signaling cost for the standard FPMIPv6, MIH-SPFP and the proposed schemes through out wireless and wired links communication. Here, as the subnet radius *r* decreases, the signaling overhead increase for all the schemes. The number of hops among the local mobility network increase with the signalling cost proportionally.

**Table 3:** Parameters list and corresponding values.

Notations	Values	Descriptions
*a*	7200m	City surface area length
*b*	4800m	City surface area width
*V*	1-50m/s	average speed of MN
*α*	2	Wireless signaling unit cost
*β*	1.5	Wired signaling unit cost
*N*	10	Average number of preferred MAGs
*τ*	20ms	Interfarme time
*T*_max_	100s	Maximum pause time in a location
$T_{{\text{L}}_{2}}$	45.35ms	Maximum pause time in a location
*P*	0.5	Probability of failures
*I*	20ms	Inter-frame time
*B*	10MHz	Bandwidth
*m*	5–20	Intermediate hops
*Z*	5–20	Neighboring networks
H_MN_MAG_	10	Distance between MN and MAG
H_MAG_MAG_	10	Distance between MAG and MAG
H_MAG_LMA_	10	Distance between MAG and LMA
H_MAG_MIIS_	10	Distance between MAG and MIIS
D_wired_	35ms	Delay over wired links
M_1_	16	Route mobility management (RS)
M_2_	64	Route Advertisement (RA)
M_3_	76	PBU
M_4_	52	PBA
M_5_	1500	MIH_Get_information request
M_6_	1500	MIH_Get_information response
M_7_	63 + 11*N+8*N*Z	MIH_Net_HO_Candidate_Query request
M_8_	77 + 101*N	MIH_Net_HO_Candidate_Query response
M_9_	75	MIH_MN_HO_Commit request
M_10_	78	MIH_MN_HO_Commit response
M_11_	150 + 11*N	MIH_N2N_HO_Query Resource request
M_12_	165	MIH_N2N_HO_Query Resource response
M_13_	213	MIH_N2N_HO_Commit request
M_14_	92	MIH_N2N_HO_Commit response
M_13*e*_	264	MIH_N2N_HO_Commit request (Ext.)
M_14*e*_	92	MIH_N2N_HO_Commit response (Ext.)
M_15_	109	MIH_N2N_HO_Complete request
M_16_	112	MIH_N2N_HO_Complete response
M_19_	78	MIH_Link_Going_down
M_20_	95	MIH_Link_Up
M_21_	75	MIH_AUTH_Wireless
M_22_	368	MIH_AUTH_Wired
M_23_	152	MIH_Net_HO_Commit request
M_4_	103	MIH_Net_HO_Commit response
M_HI_	72	HI
M_Hack_	368	ack
M_25_	196	PROPOSED_AUTH_Wireless
M_26_	572	PROPOSED_AUTH_Wired
M_27_	16	L2_HO_Triggering

As the total signaling overhead is concerned, our protocol shows an increment 5.9% over the standard FPMIPv6 and 5–7% over MIH-SPFP protocols performance. This is because much of signaling overhead of our protocol goes to wired link as the it is network only handover scheme and wired link signaling cost increases exponentially as number of hops increases.

On the other hand, Fig 9 illustrates a signal overhead of wireless Link only. As clearly stated in the introduction section of this paper, the key objective of FPMIPv6 local mobility standard is to enable the network to manage a mobility of the mobile node on the behalf of it, which is eventually to reduce computational cost, power consumption and signaling cost of the mobile node. As the signaling link of the mobile node is wireless, analyzing the wireless link only overhead is crucial.

The analysis was undertaken by varying subnet radius and number of hops, where as *β* = 2, *α* = 1.5, and failure probability *p* = 0.2 with all parameters remain the same with the initial setting. The result shows the proposed protocol is advantageous over both the standard FPMIPv6 and MIH-SPFP with an amount of 84.1% 86.9% respectively.

Fig 10 shows the handover failure probability effect on wireless link only. The wireless link failure probability varies from 0.2 to 0.65, the cell radius is set to 250m as the speed of the mobile is set to [1, 50] m/s, where as the number hops differs from 5 to 32. The signaling cost of fast handover is slightly higher than the standard handover, while the proposed solution is lower than others.

As as handover failure probability *P* increases, the residence time that the MN resides in the serving network also increases so that the MN gets more time to complete the handover process. As a result, handover delay time increase and signaling overhead also added up due to re-transmission. Though, signaling overhead increases with failure probability, the proposed scheme is far better when wireless only signaling cost in concerned (see Fig 10). Thus, the proposed scheme aligns with the objective of the local mobility management and is preferably applicable to the resource limited mobile devices. Eventually, we analyzed a signaling cost effect of wireless link while the subnet radius *r* is varied from 130m to 310m. The wireless link failure probability 0.2, the speed of the mobile is set to [1, 50] m/s, where as the number hops is 10.

As radius of the subnet decreases, the border crossing rate to another subnet (serving network) for the MN decreases. This results in the lower handoff latency and signaling overhead for the fact that number of handover requests reduce. Computationally, as radius *r* decreases, handover per unit time *E*(*N*_c_) reduces as it is a function of *r* as shown in Eqs (12)–(15) above. In particular, when referring the mobile node’s signaling overhead, the signaling cost of the proposed solution is lower than the other two as shown in Fig 11, which meets the target of the protocol design.

At last, a comparison summary of our protocol with other schemes defined for network based local mobility management (PMIPv6) is shown in Table 4 below.

**Table 4:** Security and performance comparison of handover protocols.

Comparison Factors	FPMIPv6	MIH-SPFP	Proposed
MN possibly be impersonated?	Yes	No	No
MAG possibly be impersonated?	Yes	No	No
LMA possibly be impersonated?	Yes	No	No
Can DOS be launched?	Yes	Yes	No
Can replay attack be launched?	Yes	No	No
Can Man-in-the Middle attack be launched?	Yes	No	No
Can Verifier Impersonation happen?	Yes	No	No
Is location privacy preserved?	No	No	Yes
Total handover Delay performance	Ranked 1^*st*^	Ranked 2^*nd*^	Ranked 3^*rd*^
Wireless link handover delay performance	Ranked 2^*nd*^	Ranked 3^*rd*^	Ranked 1^*st*^
Total signaling cost	Ranked 1^*st*^	Ranked 2^*nd*^	Ranked 3^*rd*^
Wireless link signaling cost	Ranked 2^*nd*^	Ranked 3^*rd*^	Ranked 1^*st*^

## 9 Conclusion

The proposed protocol is conclusively safe under formal analytical verification and efficient in the view of performance matrix analysis results. An introduction of a new pre-attachment key agreement and authentication protocols applied to MIH-enabled mobility management makes a significant enhancement in terms performance and security of the network (FPMIPv6).

As analyzed, MES-FPMIPv6 is a solution for various security requirements such as mutual authentication, key agreement, confidentiality, integrity, defence ability against compromised MAG and LMA, resistant against DoS and replay attacks. As a robust feature of our protocol, the protocol executed between MAG, LMA, MIIS and AAA server during network planning or in advance of MN handover which is to share group key and some other security credential among this entities, reduces authentication delay significantly.

Besides the above all, a concern of location privacy becomes crucial recently. MES-FPMIPv6 supports the MN’s anonymity by preserving location privacy during handover of the mobile station in a local mobility domain. We have also analyzed the proposed protocol security under BAN logic and AVISPA as its performance has been evaluated in terms of handover latency and signaling cost. As a result of these features and measures, we believe that the proposed security scheme is reasonably applicable to MIH-enabled FPMIPv6 environment. Fatherly, we would like to research inter-domain handover solution integrating FPMIPv6 with Media Independent Handover (MIH) in such a way it can mitigate security threats.

## Documentation

Analysis and avispa simulation files with plotting matlab program are deposited on figshare: https://doi.org/10.6084/m9.figshare.16438371.v1 [44].

## Supporting information

S1 Data (RAR)
